# Boron-Centered Compounds: Exploring the Optical Properties of Spiro Derivatives with Imidazo[1,5-*a*]Pyridines

**DOI:** 10.3390/molecules30122552

**Published:** 2025-06-11

**Authors:** Anita Cinco, G. Attilio Ardizzoia, Stefano Brenna, Bruno Therrien, Gioele Colombo

**Affiliations:** 1 Dipartimento di Scienza e Alta Tecnologia, Università degli Studi dell’Insubria and CIRCC, Via Valleggio, 9, 22100 Como, Italy; anita.cinco@iusspavia.it (A.C.); attilio.ardizzoia@uninsubria.it (G.A.A.); stefano.brenna@uninsubria.it (S.B.); 2 Department of Science, Technology and Society, University School for Advanced Studies IUSS, Palazzo del Broletto, Piazza Vittoria 15, 27100 Pavia, Italy; 3 Institute of Chemistry, Université de Neuchâtel, Avenue de Bellevaux 51, CH-2000 Neuchatel, Switzerland; bruno.therrien@unine.ch

**Keywords:** spiro compounds, fluorescence, blue emission, boron

## Abstract

Five boron-centered spiro compounds with imidazo[1,5-*a*]pyridin-3-yl phenols as ligands were synthesized and thoroughly characterized through ^1^H-NMR, ^13^C-NMR, infrared spectroscopy, and X-ray single crystal analysis. The fluorescence properties of these compounds in solution and in the solid state were investigated, revealing blue emission with wavelengths maxima dependent on the electronic properties of the substituents on the ligands in solution, and an orange-red emission in the solid state. Time-Dependent Density Functional Theory (TD-DFT) calculations were performed to describe the nature of the transitions

## 1. Introduction

Organic light-emitting diodes (OLEDs) have emerged as a promising display technology for future applications. However, challenges persist, particularly in achieving efficient blue emission [1] with a lack of blue emissive materials as efficient as its red and green counterparts [2].

**Figure 1:**
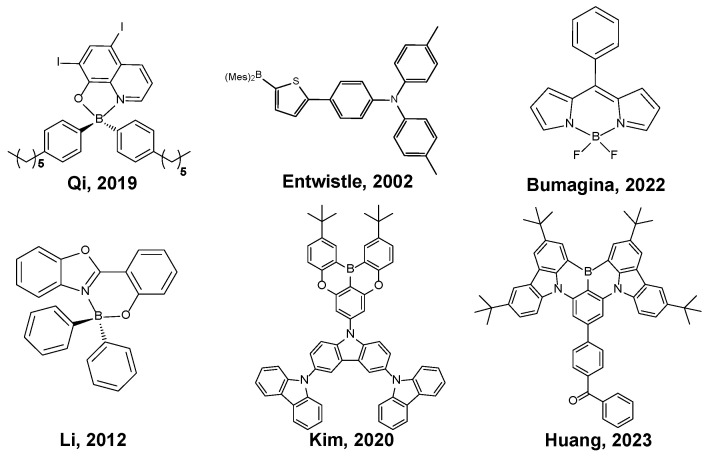
Relevant examples of organoboron luminescent compounds [6,7,8,10,11,12].

In this regard, boron-containing organic compounds have garnered attention in the field of luminescent materials due to their distinctive photophysical properties [3] (Figure 1). These compounds often exhibit blue emission and offer a versatile class of stimuli-responsive materials [4], including aggregation-induced emission (AIE) [5] and mechano-responsive luminescence [6]. The excellent photophysical characteristics of boron-containing compounds, such as a broad transparency range [7], robust thermal and chemical stability [8] and high optical damage threshold [9], make them well suited for optical materials. Furthermore, the extension of the π system of the ligands enables tunable emission across the visible spectrum [10,11]. Moreover, boron-containing compounds can serve as efficient emissive materials in OLEDs. By harnessing their unique electronic structure and tunable emission properties, boron-based emitters offer a promising avenue for achieving high-performance OLED devices with enhanced efficiency and color purity [12].

In particular, spiro-boron compounds are notable within this realm of research due to their distinctive structural characteristics, particularly the imposition of a more rigid framework by the two nearly perpendicular cyclic substructures [13,14]. This inherent rigidity contributes to unique electronic properties: the constrained molecular geometry reduces non-radiative decay pathways, consequently augmenting luminescence quantum yield [15]. Additionally, the perpendicular arrangement of the moieties often results in a significant separation of frontier orbitals, a crucial attribute for species intended to enhance optical efficiency in optoelectronic devices by leveraging mechanisms such as Thermally Activated Delayed Fluorescence (TADF) [16]. Also, such a disposition hampers the formation of π-π stacking in a molecular structure, thus favoring emission in the solid state [17].

On the other hand, imidazo[1,5-*a*]pyridines represent a class of heterocyclic compounds that have garnered significant interest [18,19] in both academic and industrial research due to their diverse pharmacological activities [20,21,22] and versatile synthetic accessibility [23,24]. Beyond their established roles as pharmaceutical agents, several investigations have unveiled intriguing photophysical properties exhibited by these compounds, particularly their fluorescence emission behavior [25,26,27,28]. Understanding the factors governing the fluorescence emission of imidazo[1,5-*a*]pyridine derivatives hold considerable importance, not only for elucidating their fundamental photophysical mechanisms but also for unlocking their potential in various technological applications such as optoelectronic devices [29], sensors [30], and imaging probes [31,32,33].

In our ongoing investigation into the coordination chemistry of *N*-based multidentate ligands [34,35,36,37], we also deeply explored differently substituted *N*,*N*- and *N*,*O*-bidentate imidazo[1,5-*a*]pyridines as potential ligands towards zinc(II), silver(I) and boron(III) centers. Unfortunately, as free species, the imidazo[1,5-*a*]pyridine phenols considered so far did not show relevant photophysical properties (i.e., fluorescence quantum yield), being almost non-emissive. Therefore, in light of the above considerations and of previous studies on boron(III) luminescent compounds [38,39,40,41,42,43,44], we elected to integrate the characteristics of these two classes, synthesizing a collection of spiro–boron compounds employing *N*,*O*-bidentate imidazo[1,5-*a*]pyridine ligands. Our focus was specifically directed toward investigating the photophysical properties arising from the unique architecture of these systems, with the goal of achieving an overall improvement over the parent imidazo[1,5-*a*]pyridines.

## 2. Results and Discussion

### 2.1. Synthesis and Characterization

**Scheme 1:**
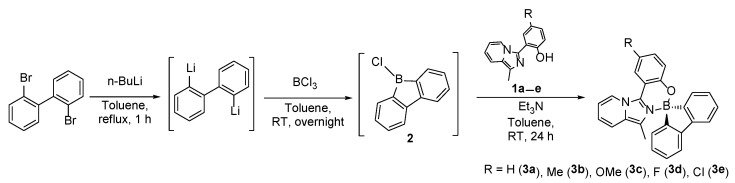
One-pot three-step synthesis of boron compounds **3a**–**e**.

The imidazo[1,5-*a*]pyridine derivatives **1a**–**e** were prepared following established procedures [39]. The synthesis of the spiro–boron compounds was accomplished through a modification of a one-pot three-steps procedure (Scheme 1) [45]. Initially, 9-chloro-9-borafluorene **2** was generated in situ by reacting 2,2′-dibromobiphenyl with *n*-butyllithium followed by reaction of the corresponding dilithium salt with boron trichloride. Subsequently, the resulting intermediate was reacted in situ with **1a**–**e** to give **3a**–**e** as orange to yellow solids, in good yields.

The products were first characterized by ^1^H and ^13^C NMR (see Supporting Information for full spectra, Figures S1–S10). The ^1^H NMR spectra of compounds **3a**–**e**, recorded in CDCl_3_, exhibited a series of signals ranging from 8.60 to 6.80 ppm ascribed to the aromatic protons of the imidazopyridine ligand and the phenyl ring of the biphenyl moiety, alongside a singlet at approximately 1.75 ppm associated with the methyl group in position 1 of the heterocycle. Correspondingly, in the ^13^C NMR spectra, resonances within the range 116–133 ppm were observed for the aromatic CH carbons, while the methyl carbon exhibited a resonance at around 10 ppm. Notably, resonances of phenylic carbon atoms directly bound to boron were absent in the spectra due to coupling with the quadrupolar boron nucleus [46].

**Figure 2:**
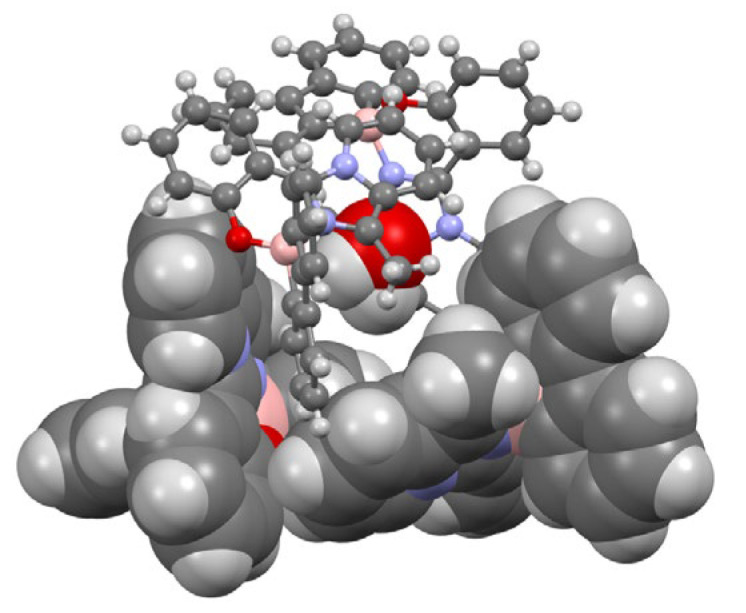
Cubic structure formed by four molecules of **3a** (two ball and stick and two space filling representations) showing a water molecule (space filling) sitting inside the cavity. Color code: dark grey: carbon atoms, light grey: hydrogen atoms, red: oxygen atoms, light blue: nitrogen atoms, pink: boron atoms.

**Figure 3:**
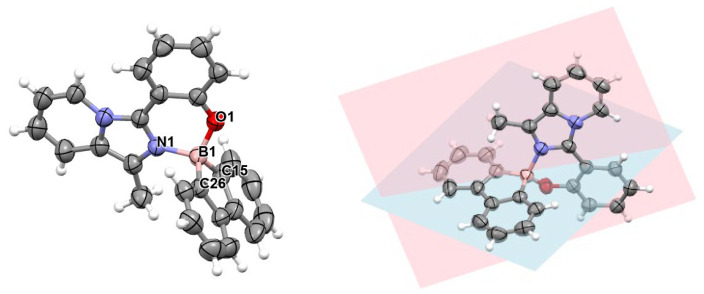
**Left**: ORTEP representation of **3a** at 50% probability level, with atom labeling. **Right**: planes described by the imidazo[1,5-*a*]pyridine moiety (pink) and the biphenyl group (light blue).

Crystals of **3a**, suitable for X-ray structure analysis, were then obtained by slow diffusion of diethyl ether in a saturated dichloromethane solution (technical grade). The compound crystallized in the tetragonal space group I 4_1_/a, showing an interesting packing in which four molecules of **3a** are positioned in a cubic shape structure (Figure 2), thus creating in the crystal packing a cavity of 652 Å^3^. In such a cavity, only small molecules can fit, typically water, that could come from adventitious water present in the crystallization solvents. Indeed, as suggested by the Mask option in Olex2 (see Section 3 for more details), the observed electron density of 152 electrons is consistent with a water molecule (expected 160 electrons). Unfortunately, in the crystal, the solvent molecule was disordered over multiple positions and was not refined. Nevertheless, the molecular structure of **3a** was confirmed by single-crystal X-ray structure analysis (Figure 3). Selected bond distances and angles are provided in Table 1, while additional crystallographic data can be found in Table S1.

As expected, the boron center exhibits a tetrahedral geometry, as corroborated by the calculated τ_4_ index (0.92) [47]. Bond lengths around the boron measure 1.592(2) Å (B-N), 1.484(2) Å (B-O), and 1.611–1.615(2) Å (B-Cphenyl), similar to values documented in the literature for bis(phenyl) borane analogues [48]. These values align precisely with those obtained for the optimized ground state (S_0_) (vide infra). The two planes delineated by the imidazo[1,5-*a*]pyridine skeleton and the biphenyl moiety exhibit a nearly perpendicular orientation, forming an angle of 77.7° (Figure 3), which is slightly less than those reported in the literature (88–90°) [14,17], possibly due to the less rigid structure of the phenol-imidazopyridine moiety when compared to other compounds with similar architectures.

The nearly perpendicular arrangement of the two cycles around the boron center hinders intermolecular π-π interactions in the crystal packing. In contrast, relevant C-H···O contacts (2.679 Å) are observed among the four molecules of **3a** in the abovementioned cubic shape structure (Figures S11 and S12). The almost total absence of π-π interactions is also confirmed by analysis of the Hirshfeld surfaces. A Hirshfeld surface analysis was conducted on the **3a** derivative. It revealed that H–H contacts were the predominant interactions, constituting 55.5% of the total lattice interactions. These interactions were observed both inside and outside the cavity, as evidenced in the fingerprint plot (Figure S13a). In particular, the diffuse and less dense region in the upper right corner of the plot is consistent with the literature [49], which attributes this pattern to interactions occurring within a small cavity. Conversely, the small spike in the lower left region represents H–H interactions between different tetramers.

The second most significant interaction type identified was H–C contacts (33.1%), with a notable contribution from C–H···π interactions. These interactions are represented by the “wings” on the sides of the fingerprint plot, as well as on the Hirshfeld surface mapped with the curvedness function (Figure S13b). The presence of yellow and red spots indicates contact points between molecules, highlighting the occurrence of intra-tetramer interactions, similar to the diffuse zone seen for H–H contacts in the top right of the fingerprint plot.

Interestingly, despite the considerable number of π-rings in the system, π–π interactions (denoted as C–C interactions in Hirshfeld analysis) accounted for only 3.3% of the total interactions. This is corroborated by the Hirshfeld surface mapped with the shape index function (Figure S13c), where the characteristic pattern associated with π–π stacking is marginal.

The surface mapped for d_e_ (Figure S13d), although uniformly covered with red spots indicating numerous interactions, provides limited additional insight, primarily confirming the high interaction density across the molecule.

Finally, an additional interaction within the cavity was identified between a hydrogen atom of a pyridine ring and an oxygen atom of a phenolic group.

Collectively, these interactions likely contribute to the significant bathochromic shift observed in the solid-state emission spectrum compared to the solution-state emission. Additionally, they may explain the presence of a minor absorption band around 500 nm in the absorption spectra recorded in the solid state (vide infra), typically associated with aggregation phenomena.

### 2.2. Optical Properties

#### 2.2.1. Analysis in Solution

**Figure 4:**
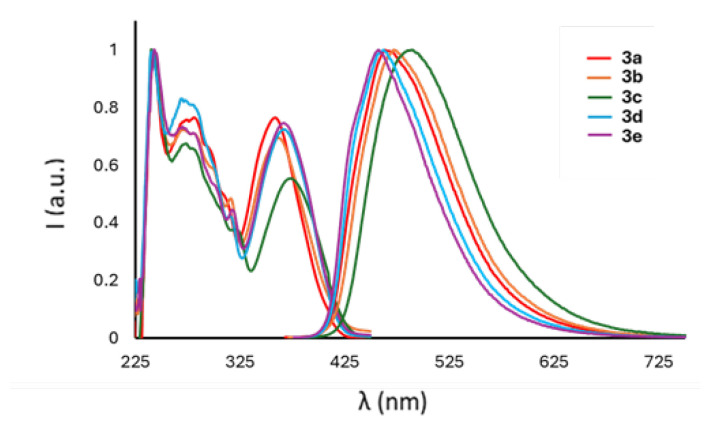
Normalized absorption and emission spectra of compounds **3a**–**e** recorded in solution (CH_2_Cl_2_, 5 × 10^−5^ M).

The absorption and emission spectra of compounds **3a**–**e** are collected in Figure 4 and Figure S14, whereas comprehensive photophysical data are compiled in Table 2 and Table S2 (Supporting Information).

The boron compounds **3a**–**e** exhibit similar absorption spectra characterized, especially in terms of absorption maxima, by a prominent absorption around 240 nm and a lower energy transition at approximately 365 nm, indicating an influence of the substituent R of the phenolic ring on the UV-visible spectra. However, distinctive variations are observed in the emission spectra: all derivatives display intense emission bands in the blue region (Table 2), revealing a discernible trend dependent on the electron properties of the substituent R. Specifically, the presence of stronger electron-donating groups leads to longer emission wavelengths. Utilizing σ_p_ Hammett constants as reliable descriptors of the substituents’ electronic properties, a trend is observed with the emission maxima (Figure S15). Notably, substituents with positive σ_p_ values (i.e., electron-withdrawing substituents) induce a hypsochromic shift, whereas those with σ_p_ values lower than zero (donor substituents) result in a slight bathochromic shift of the emission. The presence of (imidazo[1,5-*a*]pyridin-3-yl)phenolates ligands induces substantial Stokes shifts (91–116 nm, approximately 0.7–0.8 eV) across all derivatives.

Fluorescence lifetime decays are all mono-exponential, ranging from 2.7 to 3.3 ns. Fluorescence quantum yields are good when compared to other boron–spiro compounds present in the literature [48,50,51,52], with the compounds bearing electron-donating groups showcasing smaller values. Furthermore, solvent effects are deemed negligible; UV-visible, excitation, and emission spectra for **3a**, serving as a representative example, were collected in solvents with varying polarity (dichloromethane, toluene, acetonitrile, ethanol, acetone), with no significant differences observed (Figure S16 and Table S2). In contrast, boron-based spiro compounds reported in the literature exhibit pronounced solvatochromism, with emission maxima shifting by as much as 100 nm depending on the solvent polarity [52]. Moreover, their photoluminescence quantum yields can also vary considerably across different solvents. In the case of compound **3a**, however, both the emission wavelength and quantum yield remain largely unaffected by the solvent environment. This minimal solvent dependence suggests a highly rigid and electronically insulated structure, in which the excited state is weakly influenced by external forces. Such behavior is particularly advantageous for potential optoelectronic applications—such as OLEDs or fluorescence-based sensors—where stable and predictable photophysical properties across various media are essential for reliable performance.

As has been said, only fluorescence lifetimes were detected, ruling out the possibility of observing TADF, which is often seen in boron–spiro compounds [16,53]. On the other hand, the emission in such compounds can be highly dependent on the substituents—ranging from deep blue to green-yellow—[51,52]; in the species herein described, the emission window is more restricted, focusing on the blue to light-blue region of the spectrum. Interestingly, among the boron–spiro compounds family, the emission can remain virtually constant regardless of the nature of the substituents [50]. This implies that the electronic properties of the system, and consequently the excited-state behavior, can be remarkably robust. While excessive variability can be problematic—potentially shifting the emission outside the desired blue region—excessive rigidity may also be limiting, as it prevents fine-tuning of the photophysical properties. Therefore, achieving a balance between tunability and spectral stability is key for optimizing these systems toward efficient and color-pure blue emission.

#### 2.2.2. Solid State Analysis

**Figure 5:**
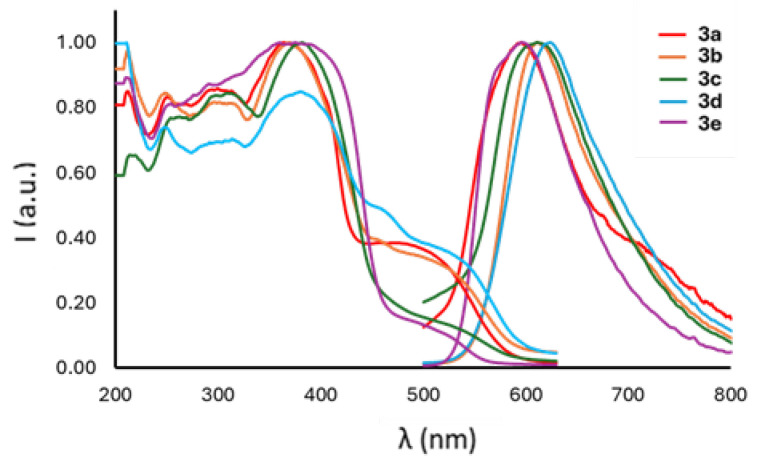
Normalized absorption and emission spectra of compounds **3a**–**e** recorded in the solid state.

The absorption and emission spectra of compounds **3a**–**e** in the solid state (powders) are collected in Figure 5.

In the solid state, all compounds exhibited absorption spectra akin to those observed in the solution, with the most intense absorption observed in the range 367–385 nm and with minimal variation depending on the substituents. An additional small shoulder at about 500 nm is detected, reasonably associated with aggregation phenomena, as already documented for spiro compounds [50]. As aforementioned, the presence of the two nearly perpendicular cycles of the spiro configuration decreases potential stacking due to π-π interactions. On the other hand, C-H···O contacts were present in the crystal packing and contributed to holding the already mentioned cubic shape structure, ultimately leading to a significant bathochromic shift observed in the solid-state emission spectrum compared to the solution [50]. For compounds **3b**–**e**, the nature of the intermolecular interactions was not investigated, but the reason for the red-shifting of the emission spectra in the solid state could still reside in some intermolecular interactions within the solid state structure.

**Figure 6:**
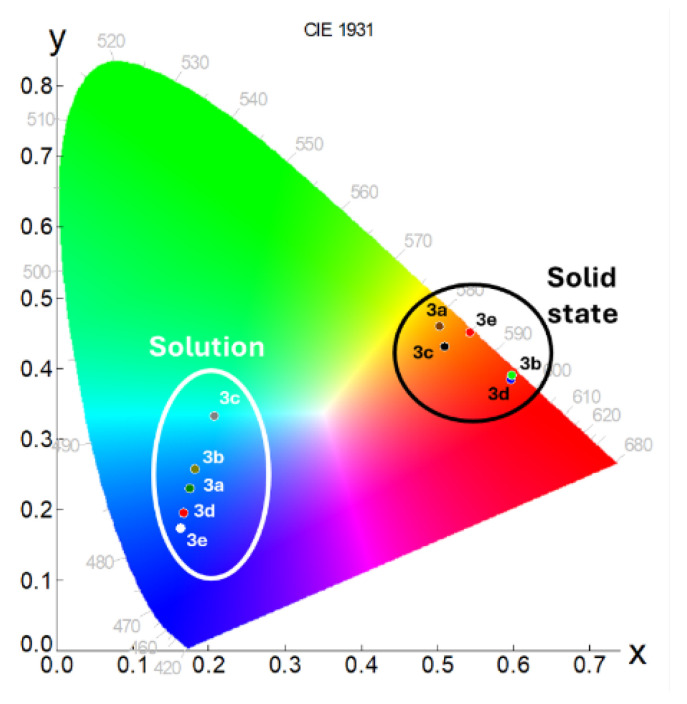
CIE 1931 chromaticity plot of compounds **3a**–**e** emissions in solution and the solid state.

Indeed, the emission maxima were consistently situated in the orange-red region of the visible spectrum (595–624 nm), accompanied by bi-exponential lifetime decays slightly shorter than those observed in solution (Table 3). As a result, a significant contribution of the solid-state interactions to the emission was observed, leading to a bathochromic shift, with respect to the solution emission, spanning the range of 120–160 nm, as depicted in the chromaticity plot below (Figure 6). Unfortunately, fluorescent quantum yields experienced a reduction compared to the one in solution (Table 3).

#### 2.2.3. UV-Vis Optical Gap

**Figure 7:**
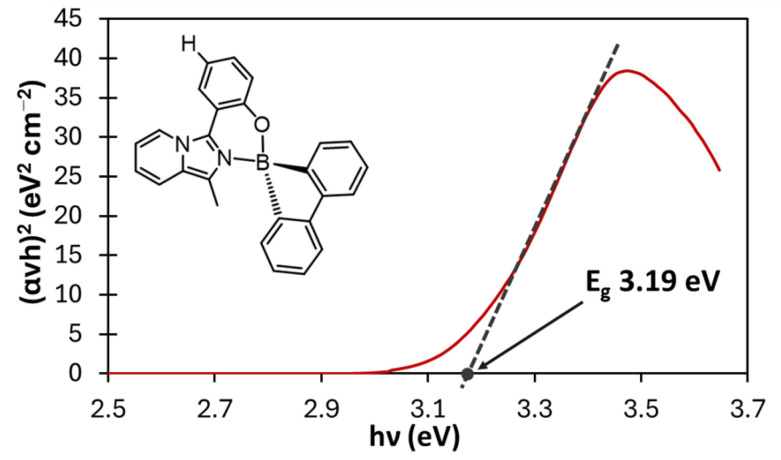
Tauc’s plot of compound **3a** (CH_2_Cl_2_, 5 × 10^−^^5^ M) with the respective band gap energy estimation.

The optical band gap (E_g_) can be assessed employing Tauc’s plot method [54,55,56], wherein E_g_ is ascertained via a linear extrapolation of the spectral dependence of (αhν)^2^ (Equation (1)). This extrapolation intersects the abscissa axis, representing the photon energies (hν), according to the following relationship:

(αhν)^r^ ∝ (hν − E_g_) (1)

where α is the absorption coefficient (evaluated as 2.303 × absorbance) [57,58], hν is the photon energy, and r depends on whether the transition considered is direct (r = 2), as in this case, or indirect (r = ½). By plotting (αhν)^2^ vs. hν, the plot reported in Figure 7 could be obtained.

The extrapolation from the linear portion of the curve goes to zero at 3.19 eV, thus giving the band gap energy for compound **3a**. Tauc’s plots of all compounds **3a**–**e** are reported in Figure S22.

### 2.3. DFT Calculations

The present study employed theoretical calculations based on Density Functional Theory (DFT) to elucidate the underlying mechanisms governing the absorption processes observed in the investigated compounds. Initially, geometry optimization utilizing the PBE0 functional was conducted, as already performed in recent studies concerning imidazo[1,5-*a*]pyridine derivatives [41].

**Figure 8:**
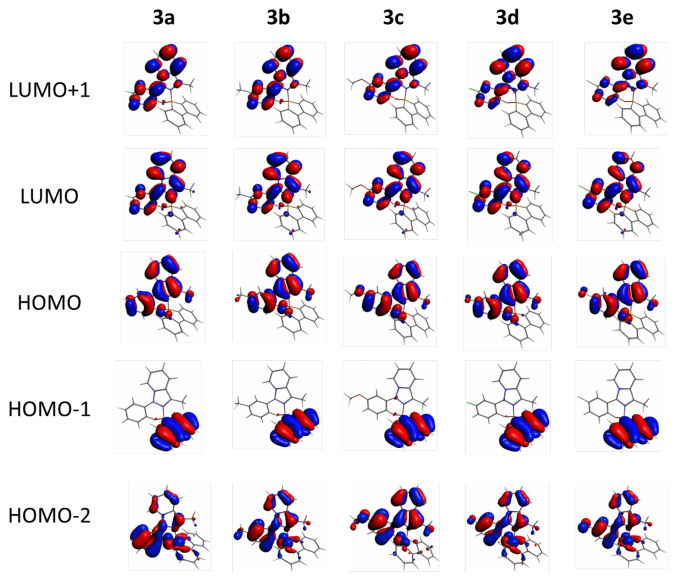
Frontier molecular orbitals of compounds **3a**–**e**.

Analysis of the frontier orbitals topology revealed consistent characteristics across all derivatives. Specifically, the LUMO+1, LUMO, and HOMO orbitals predominantly localized over the imidazopyridinyl-phenol moiety, with a minimal contribution from the boron center, which primarily serves a structural role. Conversely, HOMO-1 orbitals exhibited dispersion over the biphenyl portion of the molecules (Figure 8). Detailed information regarding the energies of these orbitals can be found in Table 4.

TD-DFT calculations were utilized to establish consistency between theoretical predictions and experimental observations.

**Figure 9:**
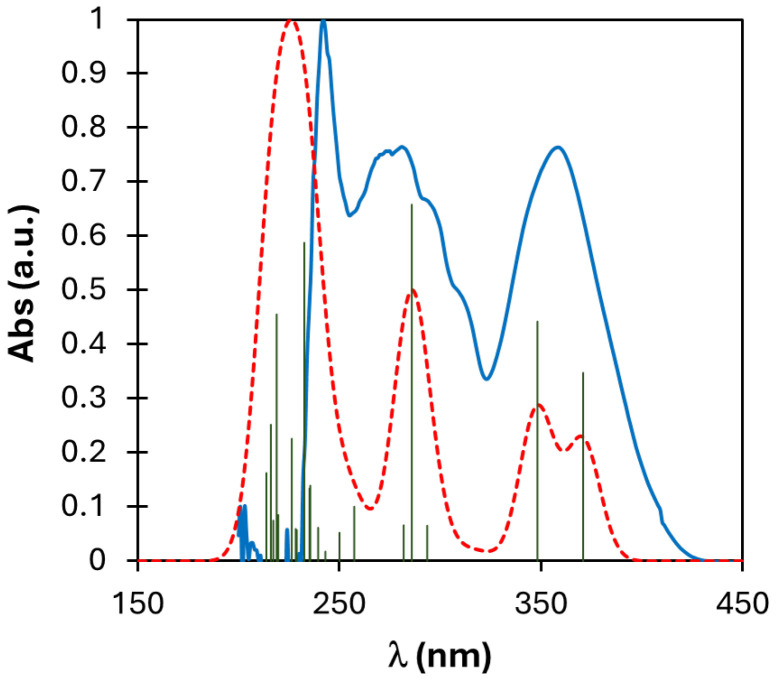
Experimental (solid blue line) and calculated (dashed red line) UV-vis spectra of compound **3a** in dichloromethane solution. Green vertical lines represent the oscillator strength associated with the electronic transition.

The UV-vis spectra of the entire series were simulated in dichloromethane solution, demonstrating a favorable agreement with the experimental data (Figure 9 and Figure S23). Analysis of the spectra revealed that the transitions primarily stemmed from HOMO-LUMO transitions (>95%), albeit with a minor contribution from other orbitals such as HOMO-1, HOMO-2, and LUMO+1 (Table 5).

However, the PBE0 functional exhibited limitations in accurately predicting the HOMO-LUMO energy gap to be compared with values obtained through Tauc’s plot from UV spectra. Consequently, in order to obtain a better agreement between calculated and experimental values, a comprehensive benchmark of various functionals was conducted (Table S3). Nonetheless, none of the assessed functionals proved to be successful in resolving this issue. Furthermore, it is well known that DFT-calculated energy gaps are not always reproducible or precise [54], in particular for what concerns an accurate determination of the LUMO energy.

This problem was circumvented by evaluating the HOMO-LUMO band gap following Equation (2):

Eg = IP − EA(2)

where IP is the ionization potential and EA the electron affinity of a compound (obtained from the energies of the neutral molecule and the corresponding radical-cation or anion) [59,60]. Again, a considerable disparity between the calculated values and the experimental data remains after employing the PBE0 function (Table 6). Optimization of the S_1_ state did not give any substantial improvement. Subsequent utilization of the PBE-D3(BJ) functional yielded more precise values (Table 6). Using this functional, it was possible to find a good accordance with the experimental data. The calculated HOMO-LUMO band gap for each compound, along with the percentage deviation from the experimental data, are detailed in Table S4.

## 3. Experimental Section

### 3.1. Materials and Methods

Infrared spectra were acquired on a Shimadzu Prestige-21 spectrometer (Kyoto, Japan) with a 1 cm^−1^ resolution; ATR spectra were acquired on a Thermo Scientific™ Nicolet™ iS20 FTIR Spectrometer (Waltham, MA, USA) with a 1 cm^−1^ resolution. Elemental analyses were obtained with a Perkin-Elmer CHN Analyzer 2400 Series II (Waltham, MA, USA). NMR spectra were recorded with an AVANCE 400 Bruker spectrometer (Billerica, MA, USA) at 400 MHz for ^1^H NMR and 100 MHz for ^13^C{^1^H}. Chemical shifts are given as δ values in ppm relative to residual solvent peaks as the internal reference. *J* values are given in Hz. The UV-vis, excitation, and emission spectra were measured using a fluorescence spectrometer (Edinburgh Instruments FS5, Livingston, UK) equipped with a 150 W continuous Xenon lamp as a light source and an additional detector for transmittance and were corrected for the wavelength response of the instrument; lifetime measurements were performed on the same FS5 Edinburgh Instruments using an EPLED-320 (Edinburgh Instruments) as the pulsed source. Analysis of the lifetime decay curve and determination of absolute quantum yields were carried out using Fluoracle^®^ Software package (Ver. 1.9.1) which runs the FS5 instrument. Absolute fluorescence quantum yields in the solid state were determined on a Photon Technology International (PTI) QuantaMaster QM-40 spectrometer (Xe arc lamp, 70 W) (Photon Technology International, Brimingham, NJ, USA) with a PhotoMed GmbH K-Sphere Integrating Sphere (3.2 inch. diameter) (Photon Technology International, Brimingham, NJ, USA). UV-vis spectra in the solid state were measured using a Shimadzu UV-2600 spectrophotometer. All chemicals were of reagent grade quality and were purchased commercially (AlfaAesar (Haverhill, MA, USA), Acros (Geel, Belgium), TCI Chemicals (Tokyo, Japan), Fluorochem (Derbyshire, UK)) and used as received.

### 3.2. General Procedure for the Synthesis of Boron Compounds ***3a***–***e***

2,2′-Dibromobiphenyl (1.5 g, 4.8 mmol, 1 eq) was dissolved in 30 mL of degassed toluene. Subsequently, 6.25 mL of *n*-butyllithium (1.6 M in hexane, 10 mmol, 2 eq) were slowly added dropwise, at 0 °C. The resultant mixture was then refluxed for 1 h, resulting in the precipitation of a white solid. After cooling to 0 °C, 5 mL of BCl_3_ (1.0 M in hexane, 5 mmol, 1 eq) were added dropwise. The temperature was allowed to rise to room temperature, and the mixture was stirred overnight. A solution containing the desired imidazo[1,5-*a*]pyridine-3-yl phenol (1 eq) and 668 μL (4.8 mmol, 1 eq) of triethylamine in 30 mL of degassed toluene was then added dropwise. The mixture was allowed to react at room temperature for 24 h. The resulting suspension was filtered, and the solids were discarded. The solvent was then removed from the filtrate under reduced pressure at room temperature to yield orange solids.

**3a**: Yield: 1.19 g (64%). Anal. Calcd. (%) for C_26_H_19_N_2_OB: C, 80.85; H, 4,96; N, 7.25. Found (%): C, 80.92; H, 5.33; N, 6.88. ^1^H NMR (400 MHz, CDCl_3_, 298 K, *J* [Hz]): δ = 8.57 (d, *J* = 7.0, 1H), 7.89 (d, *J* = 7.04, 1H), 7.67 (d, *J* = 7.52, 2H), 7.37 (t, *J* = 7.82, 2H), 7.26 (m, 2H), 7.19–7.17 (m, 3H), 7.09–7.01 (m, 3H), 6.88–6.81 (m, 2H), 1.77 (s, 3H). ^13^C NMR (101 MHz, CDCl_3_, 298 K): δ = 159.7, 148.9, 132.0, 131.9, 129.9, 128.1, 127.7, 126.7, 123.4, 122.1, 122.1, 121.3, 119.8, 119.5, 119.0, 118.9, 116.5, 113.2, 10.0.

**3b**: Yield: 1.34 g (70%). Anal. Calcd. (%) for C_27_H_21_N_2_OB: C, 81.02; H, 5.29; N, 7.00. Found (%): C, 81.13; H, 5.18; N, 6.76. ^1^H NMR (400 MHz, CDCl_3_, 298 K, *J* [Hz]): δ = 8.65 (d, *J* = 7.2 Hz, 1H), 7.74 (d, *J* = 7.6 Hz, 3H), 7.42 (d, *J* = 9.0 Hz, 1H), 7.34 (t, *J* = 8.0 Hz, 2H), 7.29–7.24 (m, 3H), 7.20–7.14 (m, 1H), 7.11 (t, *J* = 7.1 Hz, 2H), 6.91 (m, 2H), 2.55 (s, 3H), 1.84 (s, 3H). ^13^C NMR (101 MHz, CDCl_3_): δ = 157.4, 148.8, 132.9, 132.0, 130.0, 128.1, 128.0, 127.6, 126.6, 123.4, 122.2, 122.1, 121.0, 119.7, 119.4, 118.9, 116.4, 113.0, 21.2, 10.0.

**3c**: Yield: 1.23 g (62%). Anal. Calcd. (%) for C_27_H_21_N_2_O_2_B: C, 77.90; H, 5.08; N, 6.73. Found (%): C, 77.51; H, 5.00; N, 6.31. ^1^H NMR (400 MHz, CDCl_3_, 298 K, *J* [Hz]): δ = 8.43 (d, *J* = 7.0 Hz, 1H), 7.55 (d, *J* = 7.5 Hz, 2H), 7.31 (d, *J* = 2.8 Hz, 1H), 7.22 (t, *J* = 7.6 Hz, 1H), 7.14 (t, *J* = 7.4 Hz, 2H), 7.08 (d, *J* = 7.0 Hz, 2H), 7.02 (d, *J* = 8.9 Hz, 1H), 6.95–6.86 (m, 3H), 6.76–6.66 (m, 2H), 3.80 (s, 3H), 1.66 (s, 3H). ^13^C NMR (101 MHz, CDCl_3_) δ = 153.5, 152.3, 148.8, 131.6, 129.9, 128.0, 127.8, 126.6, 123.6, 121.9, 121.6, 119.9, 119.4, 118.9, 117.0, 116.6, 113.4, 108.2, 56.2, 10.0.

**3d**: Yield: 1.27 g (66%). Anal. Calcd. (%) for C_26_H_18_N_2_OBF: C, 77.25; H, 4.49; N, 6.93. Found (%): C, 76.86; H, 4.10; N, 6.64. ^1^H NMR (400 MHz, CDCl_3_, 298 K, *J* [Hz]): δ = 8.50 (d, *J* = 7.2 Hz, 1H), 7.65 (d, *J* = 7.5 Hz, 2H), 7.57 (m, 1H), 7.38 (d, *J* = 9.0 Hz, 1H), 7.29–7.21 (m, 2H), 7.20–7.07 (m, 4H), 7.01 (t, *J* = 7.1 Hz, 2H), 6.89 (m, 2H), 1.76 (s, 3H). ^13^C NMR (101 MHz, CDCl_3_, 298 K): δ = 156.8, 155.5, 148.6, 130.8, 129.8, 128.0, 127.9, 126.6, 123.8, 121.6, 120.1, 119.4, 118.9, 118.6, 118.4, 116.9, 112.9, 108.0, 9.9.

**3e**: Yield: 1.23 g (62%). Anal. Calcd. (%) for C_26_H_18_N_2_OBCl: C, 74.23; H, 4.31; N, 6.66. Found (%): C, 74.34; H, 4.76; N, 6.32. ^1^H NMR (400 MHz, CDCl_3_, 298 K, *J* [Hz]): δ = 8.53 (d, *J* = 7.2 Hz, 1H), 7.83 (s, 1H), 7.65 (d, *J* = 7.5 Hz, 2H), 7.38 (d, *J* = 9.1 Hz, 1H), 7.34–7.21 (m, 3H), 7.20–7.13 (m, 2H), 7.10 (d, *J* = 8.8 Hz, 1H), 7.02 (t, *J* = 7.1 Hz, 2H), 6.93 (t, *J* = 6.6 Hz, 1H), 6.90–6.82 (m, 1H), 1.76 (s, 3H). ^13^C NMR (101 MHz, CDCl_3_, 298 K): δ = 158.1, 148.7, 131.6, 130.4, 129.9, 128.2, 128.1, 126.7, 123.9, 123.7, 122.4, 121.8, 121.6, 120.3, 119.5, 118.9, 117.1, 114.2, 10.0.

### 3.3. X-Ray Characterization

A crystal of **3a**, grown by slow diffusion of diethyl ether and dichloromethane (technical grade) over a period of 7 days, was mounted on a STOE STADIVARI Eulerian 4-circle diffractometer (Darmstadt, Germany) equipped with a Pilatus300K detector (Dectris, Baden, Switzerland), using Cu-Kα radiation (λ = 1.54186 Å). The structure was solved and refined by direct methods using the OLEX2 platform [61]. The H-atoms were included in calculated positions and treated as riding atoms, while the non-H atoms were refined anisotropically, using weighted full-matrix least-square on *F*^2^. Crystallographic details are summarized in Table S1. Figure 2 and Figure 3 were drawn with Mercury [62]. A solvent void was observed in **3a** and estimated to be of 152 electrons in a volume of 652 Å^3^. This was consistent with the presence of one H_2_O per asymmetric unit, which normally accounts for 160 electrons per unit cell. Therefore, this void was considered to be occupied by a highly disordered water molecule, and it was removed during refinement using the Mask algorithm from OLEX2.

CCDC-2360455 (**3a**) contains the supplementary crystallographic data for this paper. These data can be obtained free of charge via www.ccdc.cam.ac.uk/data_request/cif (accessed on 7 June 2025), by e-mailing data_request@ccdc.cam.ac.uk, or by contacting The Cambridge Crystallographic Data Centre, 12, Union Road, Cambridge CB2 1EZ, UK; fax: +44 1223 336033.

### 3.4. Computational Details

All calculations were carried out at the density functional (DFT) level of theory with the ADF2022.101 program package [63,64,65,66]. The PBE0 and PBE-D3(BJ) functionals were employed for all calculations and benchmark on band gaps evaluation (see Section 2.3). Frequency analyses were performed for all optimized structures to establish the nature of the stationary points. TD-DFT implemented in the ADF package was used to determine the excitation energies: the 30 lowest singlet–singlet excitations were calculated by using the optimized geometries. For geometry optimizations B, C, N, O, Cl, and F atoms were described through TZ2P basis sets [triple-ξ Slater-type orbitals (STOs) plus two polarization functions], while H atoms were described through TZP [triple-ξ Slater-type orbitals (STOs) plus one polarization function]. The corresponding augmented basis set was employed in TD-DFT calculations [63]. Restricted formalism, no-frozen-core approximation (all-electron) and no-symmetry constraints were used in all calculations. Solvent effects (CH_2_Cl_2_) were simulated employing the conductor-like continuum solvent model (COSMO) [67,68,69] as implemented in the ADF suite.

## 4. Conclusions

In conclusion, we successfully synthesized and characterized five boron-based spiro compounds incorporating (imidazo[1,5-*a*]pyridine-3-yl)phenolate ligands. By introducing substituents with varying electronic properties (as indicated by Hammett’s constants) at the *para* position to the hydroxyl group on the phenolic ring, we achieved modulation of the fluorescence emission in solution. In addition, the insertion of a biphenyl substituent orthogonal to the imidazo[1,5-*a*]pyridine phenol moiety increased the luminescence performance of the parent compounds. All compounds exhibited favorable photophysical properties, including blue emission, large Stokes shifts, and good fluorescence quantum yields in solution, while an orange-red emission due to intermolecular C-H···O interactions was observed in the solid state. TD-DFT calculations were performed to elucidate the nature of the electronic transitions involved in the absorption processes. These calculations revealed contributions not only from the frontier orbitals but also from HOMO-1, HOMO-2, and LUMO+1.

## Figures and Tables

**Table 1:** Selected bond distances and angles for **3a**.

	Distances (Å)		Angles (°)
		N(1)-B(1)-O(1)	105.8 (1)
B(1)-N(1)	1.592 (2)	N(1)-B(1)-C(15)	115.3 (1)
B(1)-O(1)	1.484 (2)	N(1)-B(1)-C(26)	110.4 (1)
B(1)-C(15)	1.611 (2)	O(1)-B(1)-C(15)	110.4 (1)
B(1)-C(26)	1.615 (2)	O(1)-B(1)-C(26)	114.8 (1)
		C(15)-B(1)-C(26)	100.5 (1)

**Table 2:** Photophysical data for compounds **3a**–**e** recorded in dichloromethane solution (5 × 10^−5^ M).

	R	σ_p_	λ_abs_ (nm)	λ_exc_ (nm)	λ_em_ (nm)	Stokes Shift (cm^−1^)	Stokes Shift (eV)	Φ_PL_	τ (ns)	k_r_ (10^7^ s^−1^)	k_nr_ (10^8^ s^−1^)	ε (M^−1^ cm^−1^)	Optical Band Gap (eV)
**3a**	H	0	358	357	463	6533	0.81	0.20	3.2	6.3	2.5	15,523	3.19
**3b**	Me	−0.17	361	364	472	6613	0.82	0.12	2.7	4.4	3.3	15,077	3.13
**3c**	OMe	−0.27	373	372	489	7017	0.87	0.13	2.8	4.6	3.1	10,165	3.01
**3d**	F	0.06	367	367	462	5726	0.71	0.22	3.3	6.7	2.4	15,049	3.07
**3e**	Cl	0.23	366	369	457	5484	0.68	0.23	3.1	7.4	2.5	16,499	3.06

**Table 3:** Photophysical data for compounds **3a**–**e** recorded in the solid state.

	R	σ_p_	λ_abs_ (nm)	λ_exc_ (nm)	λ_em_ (nm)	Stokes Shift (cm^−1^)	Stokes Shift (eV)	Φ_PL_	τ (ns)
**3a**	H	0	367	416	595	10,441	1.29	<0.05	2.09 (80.52%) 3.66 (19.48%)
**3b**	Me	−0.17	368	532	612	10,834	1.34	<0.05	2.16 (52.52%) 3.80 (47.48%)
**3c**	OMe	−0.27	387	529	611	9473	1.17	<0.05	3.06 (81.69%) 6.54 (18.31%)
**3d**	F	0.06	385	533	624	9948	1.23	<0.05	1.68 (57.51%) 3.56 (42.49%)
**3e**	Cl	0.23	381	502	596	9468	1.17	<0.05	0.46 (96.13%) 2.35 (3.87%)

**Table 4:** Calculated orbitals’ energies (eV) of compounds **3a**–**e** (DFT//PBE0).

	3a	3b	3c	3d	3e
LUMO+1	−1.40	−1.39	−1.42	−1.48	−1.49
LUMO	−1.60	−1.59	−1.62	−1.70	−1.71
HOMO	−5.83	−5.76	−5.72	−5.85	−5.88
HOMO-1	−6.19	−6.18	−6.18	−6.21	−6.21
HOMO-2	−6.70	−6.58	−6.36	−6.68	−6.69

**Table 5:** Percentage contribution of the different transitions to lower energy absorption.

**3a**	HOMO → LUMO	86.3%
	HOMO-1 → LUMO	5.2%
	HOMO-2 → LUMO	4.9%
	HOMO → LUMO+1	1.7%
**3b**	HOMO → LUMO	89.0%
	HOMO-2 → LUMO	5.2%
	HOMO → LUMO+1	2.5%
	HOMO-1 → LUMO	1.5%
**3c**	HOMO → LUMO	88.4%
	HOMO → LUMO+1	5.7%
	HOMO-2 → LUMO	4.0%
**3d**	HOMO → LUMO	79.0%
	HOMO → LUMO+1	8.7%
	HOMO-1 → LUMO	6.3%
	HOMO-2 → LUMO	3.4%
	HOMO-2 → LUMO+1	1.6%
**3e**	HOMO → LUMO	81.0%
	HOMO → LUMO+1	6.8%
	HOMO-1 → LUMO	5.8%
	HOMO-2 → LUMO	4.0%
	HOMO-2 → LUMO+1	1.5%

**Table 6:** Calculated HOMO-LUMO band gap (eV) of compound **3a** using Equation (2).

	SP ^1^	Relaxed ^2^	Relaxed ^2^
Optical Eg	PBE0 (IP/EA)	PBE0 (IP/EA)	PBE-D3(BJ) (IP/EA)
3.19	3.82	3.46	3.15
error %	19.7	8.5	−1.3

^1^ Band gap calculated without optimizing the geometry of cation and anion. ^2^ Band gap calculated after optimization of the cation and anion geometry through the corresponding functional.

## Data Availability

Dataset available on request from the authors.

## Supplementary Materials

The following supporting information can be downloaded at: https://www.mdpi.com/article/10.3390/molecules30122552/s1, Figures S1–S10: ^1^H and ^13^C NMR spectra of compounds **3a**–**e**; Figure S11: C-H···O contacts among four molecules of **3a** in the crystal packing; Figure S12: Crystal packing of **3a** seen from other orientations; Figure S13: Fingerprint plot and Hirshfeld surfaces of 3a; Figure S14: Normalized absorption, emission and excitation spectra of compounds **3a**–**e** recorded in dichloromethane solution (5 × 10^−5^ M); Figure S15: Correlation between fluorescence emission in solution and σ_p_ Hammett’s constant of substituent R; Figure S16: Normalized absorption, emission and excitation spectra of compound **3a** recorded in various solvents (5 × 10^−5^ M); Figures S17–S21: Infrared (ATR) spectra of compounds **3a**–**e**; Figure S22: Tauc’s plots of compounds 3a–e (CH_2_Cl_2_, 5 × 10^−5^ M) with the respective band gap energy estimation; Figure S23: Calculated UV-vis spectra of compounds **3a**–**e**; Table S1: Crystallographic and structure refinement parameters for compound **3a**; Table S2: Photophysical data of compound **3a** recorded in various solvents (5 × 10^−5^ M); Table S3: Benchmark DFT calculations for compound **3a**’s HOMO-LUMO band gap; Table S4: HOMO-LUMO band gap of compounds **3a**–**e** calculated from the ionization potential and electron affinity.
