# Supplementary material for: Boron-Centered Compounds: Exploring the Optical Properties of Spiro Derivatives with Imidazo[1,5-a]Pyridines
Source: Molecules. 2025 Jun 11;30(12):2552. doi: 10.3390/molecules30122552 (PMC12195987; doi:10.3390/molecules30122552)
Supplement: Supplementary file 1 [file molecules-30-02552-s001.zip › molecules-3681981_supporting.pdf]

molecules

MDPI

Article

Boron-Centered Compounds: Exploring the Optical Properties of Spiro Derivatives with Imidazo[1,5-*a*]Pyridines

Anita Cinco [1,2], G. Attilio Ardizzoia [1], Stefano Brenna [1], Bruno Therrien [3] and Gioele Colombo [1,*]

[1] Dipartimento di Scienza e Alta Tecnologia, Università degli Studi dell'Insubria and CIRCC, Via Valleggio, 9, 22100 Como, Italy; anita.cinco@iusspavia.it (A.C.); attilio.ardizzoia@uninsubria.it (G.A.A.); stefano.brenna@uninsubria.it (S.B.)

[2] Department of Science, Technology and Society, University School for Advanced Studies IUSS, Palazzo del Broletto, Piazza Vittoria 15, 27100 Pavia, Italy

[3] Institute of Chemistry, Université de Neuchâtel, Avenue de Bellevaux 51, CH-2000 Neuchatel, Switzerland; bruno.therrien@unine.ch

* Correspondence: gioele.colombo@uninsubria.it

Contents

Figure S1-S10 ^{1}H and ^{13}C NMR spectra of compounds **3a-e**.

Figure S11 C-H···O contacts among four molecules of **3a** in the crystal packing.

Figure S12 Crystal packing of **3a** seen from other orientations.

Figure S13 Fingerprint plot and Hirshfeld surfaces of **3a**

Figure S14 Normalized absorption, emission and excitation spectra of compounds **3a-e** recorded in dichloromethane solution ($5 \cdot 10^{-5}$ M).

Figure S15 Correlation between fluorescence emission in solution and σ_p Hammett's constant of substituent R.

Figure S16 Normalized absorption, emission and excitation spectra of compound **3a** recorded in various solvents ($5 \cdot 10^{-5}$ M).

Figure S17-21 Infrared (ATR) spectra of compounds **3a-e**.

Figure S22 Tauc's plots of compounds **3a-e** (CH_2Cl_2, $5 \cdot 10^{-5}$ M) with the respective band gap energy estimation.

Figure S23 Calculated UV-vis spectra of compounds **3a-e**.

Table S1 Crystallographic and structure refinement parameters for compound **3a**.

Table S2 Photophysical data of compound **3a** recorded in various solvents ($5 \cdot 10^{-5}$ M).

Table S3 Benchmark DFT calculations for compound **3a**'s HOMO-LUMO band gap.

Table S4 HOMO-LUMO band gap of compounds **3a-e** calculated from the ionization potential and electron affinity.

 https://doi.org/10.3390/molecules30122552

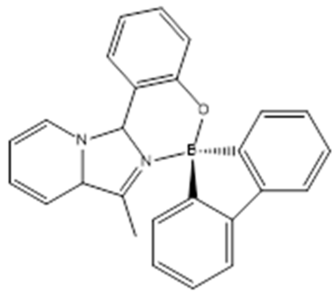

3a: ^{1}H NMR (400 MHz, $CDCl_3$, 298 K, *J* [Hz]): δ = 8.57 (d, *J* = 7.0, 1H), 7.89 (d, *J* = 7.04, 1H), 7.67 (d, *J* = 7.52, 2H), 7.37 (t, *J* = 7.82, 2H), 7.26 (m, 2H), 7.19 – 7.17 (m, 3H), 7.09 – 7.01 (m, 3H), 6.88 – 6.81 (m, 2H), 1.77 (s, 3H). ^{13}C NMR (101 MHz, $CDCl_3$, 298 K): δ = 159.7, 148.9, 132.0, 131.9, 129.9, 128.1, 127.7, 126.7, 123.4, 122.1, 122.1, 121.3, 119.8, 119.5, 119.0, 118.9, 116.5, 113.2, 10.0.

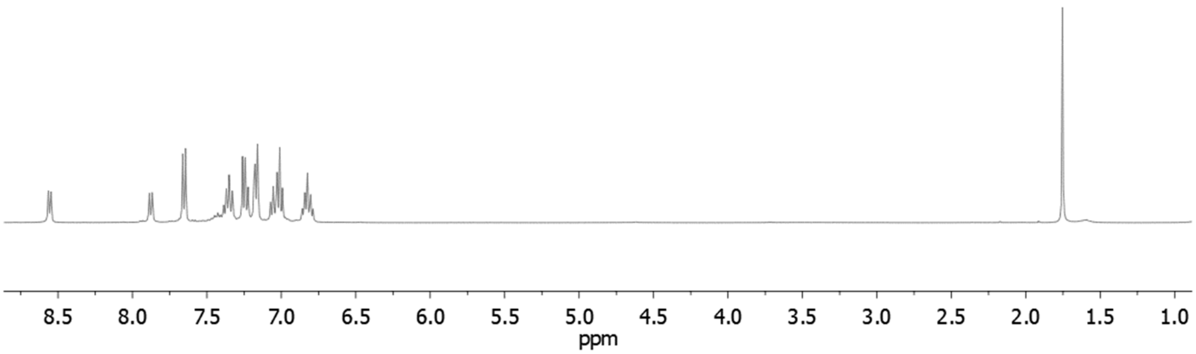

Figure S1: ^{1}H NMR spectrum ($CDCl_3$, 298 K) of **3a**.

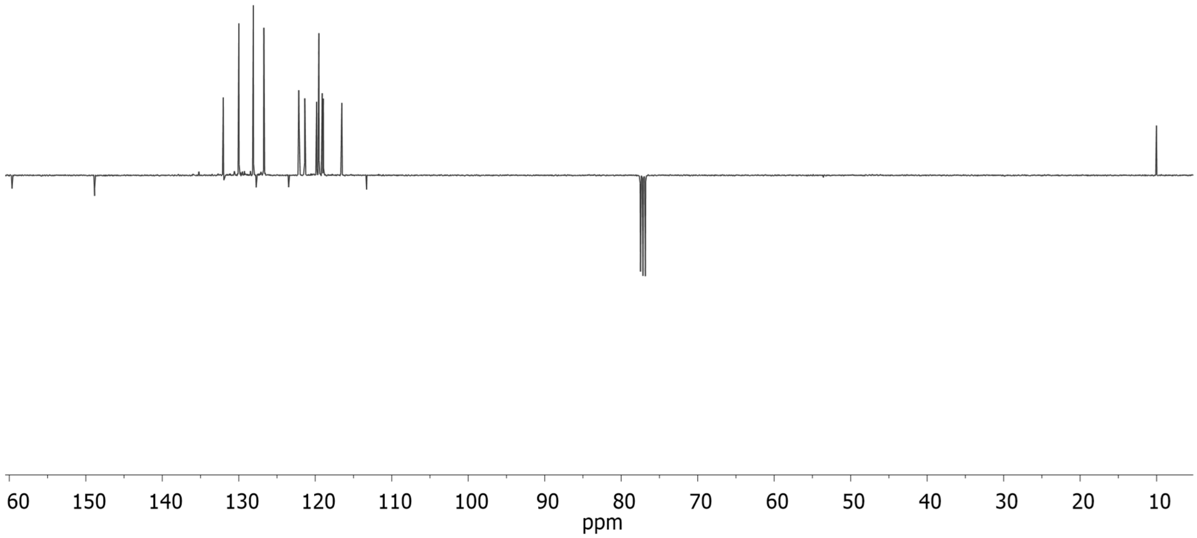

Figure S2: ^{13}C NMR spectrum ($CDCl_3$, 298 K) of **3a**.

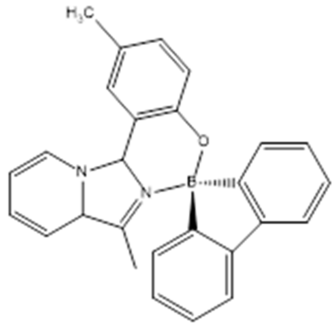

3b: ^{1}H NMR (400 MHz, CDCl$_3$, 298 K, *J* [Hz]): δ = ^{1}H NMR (400 MHz, CDCl$_3$) δ 8.65 (d, *J* = 7.2 Hz, 1H), 7.74 (d, *J* = 7.6 Hz, 3H), 7.42 (d, *J* = 9.0 Hz, 1H), 7.34 (t, *J* = 8.0 Hz, 2H), 7.29 – 7.24 (m, 3H), 7.20 – 7.14 (m, 1H), 7.11 (t, *J* = 7.1 Hz, 2H), 6.91 (m, 2H), 2.55 (s, 3H), 1.84 (s, 3H).^{13}C NMR (101 MHz, CDCl$_3$): δ = 157.46, 148.84, 132.94, 132.09, 130.01, 128.19, 128.03, 127.63, 126.67, 123.40, 122.22, 122.17, 121.08, 119.71, 119.49, 118.91, 116.41, 113.02, 21.22, 10.05.

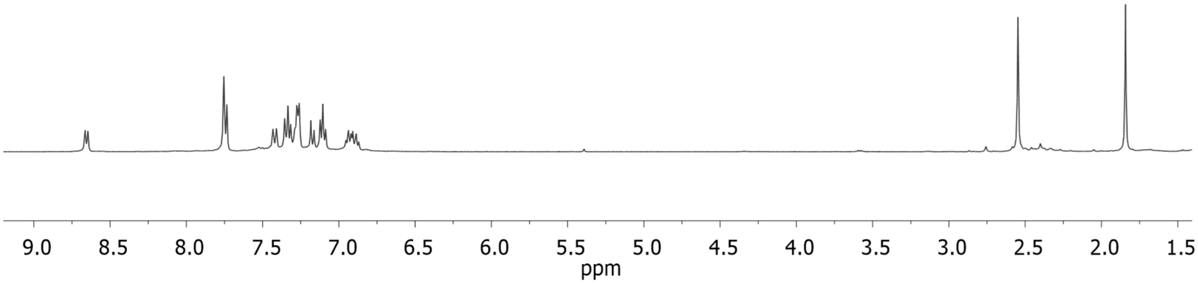

Figure S3: ^{1}H NMR spectrum (CDCl$_3$, 298 K) of **3b**.

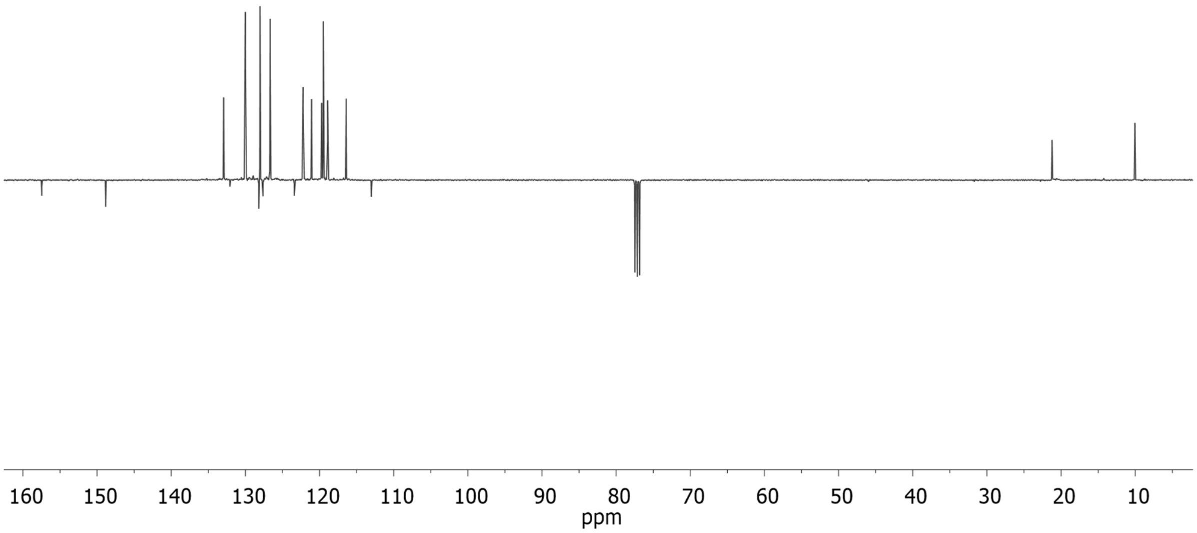

Figure S4: ^{13}C NMR spectrum (CDCl$_3$, 298 K) of **3b**.

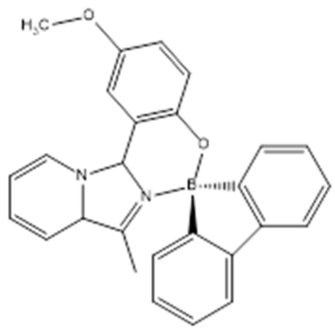

3c: ^{1}H NMR (400 MHz, $CDCl_3$, 298 K, *J* [Hz]): δ = ^{1}H NMR (400 MHz, $CDCl_3$) δ 8.43 (d, *J* = 7.0 Hz, 1H), 7.55 (d, *J* = 7.5 Hz, 2H), 7.31 (d, *J* = 2.8 Hz, 1H), 7.22 (t, *J* = 7.6 Hz, 1H), 7.14 (t, *J* = 7.4 Hz, 2H), 7.08 (d, *J* = 7.0 Hz, 2H), 7.02 (d, *J* = 8.9 Hz, 1H), 6.95 – 6.86 (m, 3H), 6.76 – 6.66 (m, 2H), 3.80 (s, 3H), 1.66 (s, 3H).^{13}C NMR (101 MHz, $CDCl_3$) δ = 153.59, 152.38, 148.83, 131.67, 129.99, 128.03, 127.80, 126.66, 123.61, 121.92, 121.68, 119.91, 119.48, 118.91, 117.07, 116.63, 113.43, 108.27 56.2, 10.0.

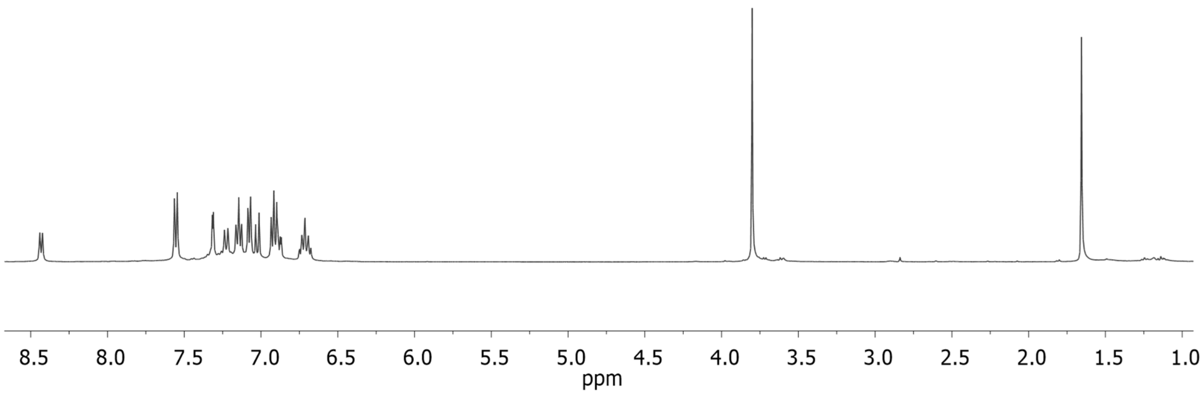

Figure S5: ^{1}H NMR spectrum ($CDCl_3$, 298 K) of **3c**.

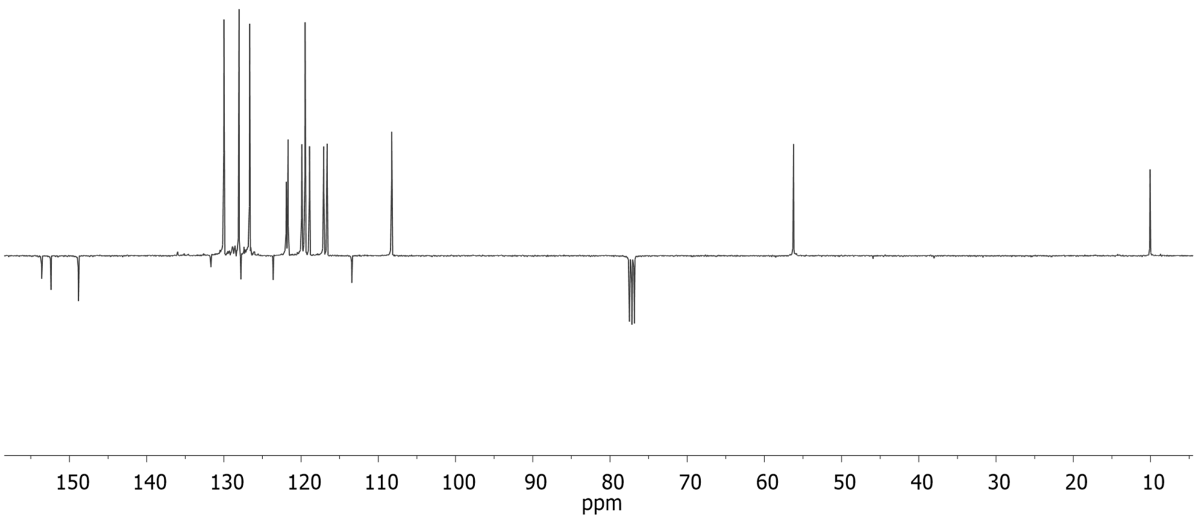

Figure S6: ^{13}C NMR spectrum ($CDCl_3$, 298 K) of **3c**.

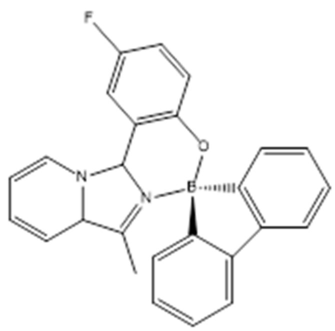

3d: ^{1}H NMR (400 MHz, $CDCl_3$, 298 K, *J* [Hz]): δ = 8.50 (d, *J* = 7.2 Hz, 1H), 7.65 (d, *J* = 7.5 Hz, 2H), 7.57 (m, 1H), 7.38 (d, *J* = 9.0 Hz, 1H), 7.29 – 7.21 (m, 2H), 7.20 – 7.07 (m, 4H), 7.01 (t, *J* = 7.1 Hz, 2H), 6.89 (m, 2H), 1.76 (s, 3H). ^{13}C NMR (101 MHz, $CDCl_3$, 298 K, *J* [HZ]): δ = 156.88, 155.59, 148.61, 130.80, 129.81, 128.09, 127.95, 126.63, 123.84, 121.63, 120.10, 119.47, 118.93, 118.69, 118.46, 116.93, 112.92, 108.06, 9.93.

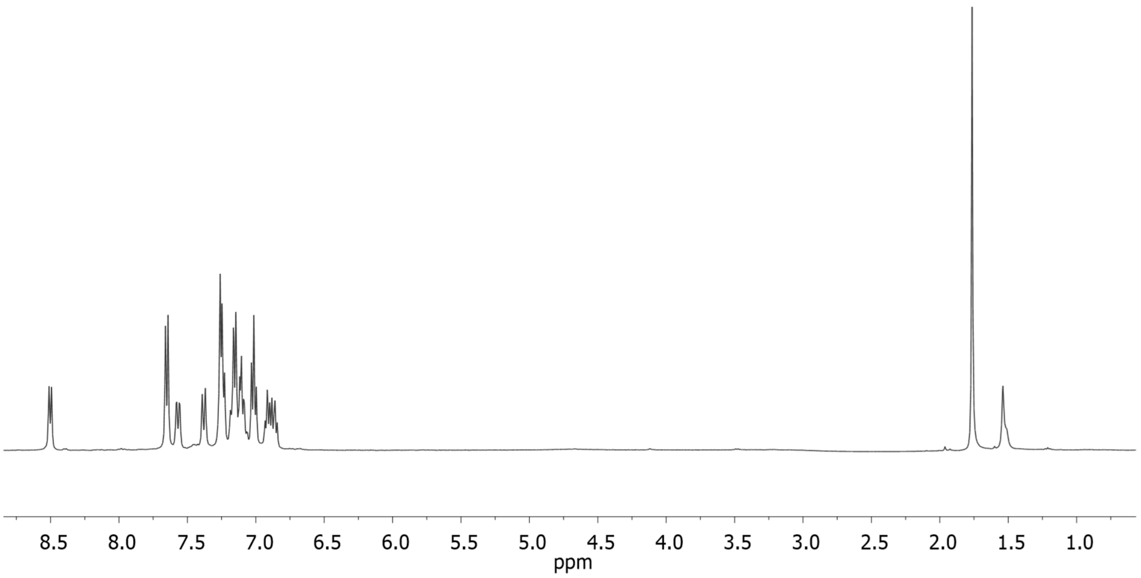

Figure S7: ^{1}H NMR spectrum ($CDCl_3$, 298 K) of **3d**.

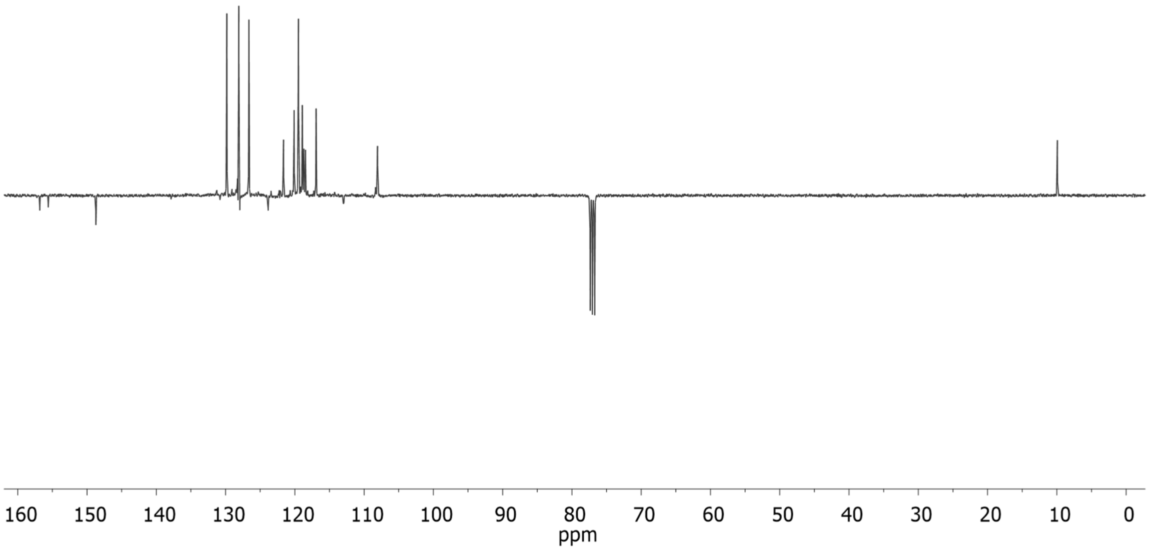

Figure S8: ^{13}C NMR spectrum ($CDCl_3$, 298 K) of **3d**.

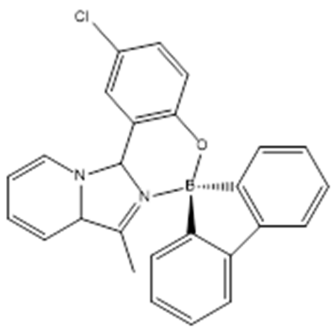

3e: ^{1}H NMR (400 MHz, $CDCl_3$, 298 K, *J* [Hz]): δ = 8.53 (d, *J* = 7.2 Hz, 1H), 7.83 (s, 1H), 7.65 (d, *J* = 7.5 Hz, 2H), 7.38 (d, *J* = 9.1 Hz, 1H), 7.34 – 7.21 (m, 3H), 7.20 – 7.13 (m, 2H), 7.10 (d, *J* = 8.8 Hz, 1H), 7.02 (t, *J* = 7.1 Hz, 2H), 6.93 (t, *J* = 6.6 Hz, 1H), 6.90 – 6.82 (m, 1H), 1.76 (s, 3H). ^{13}C NMR (101 MHz, $CDCl_3$, 298 K, *J* [Hz]): δ = 158.10, 148.78, 131.64, 130.43, 129.92, 128.23, 128.10, 126.76, 123.93, 123.72, 122.49, 121.85, 121.60, 120.32, 119.57, 118.94, 117.13, 114.27, 10.00.

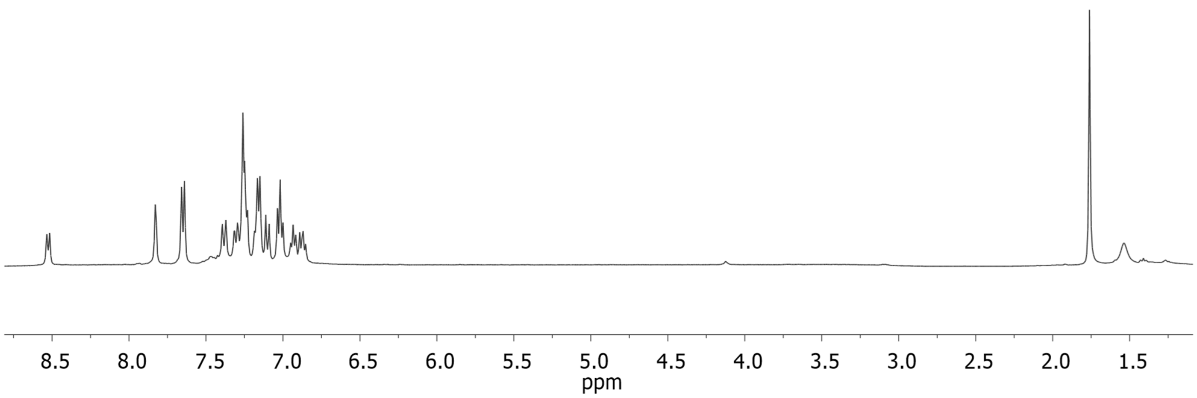

Figure S9: ^{1}H NMR spectrum ($CDCl_3$, 298 K) of **3e**.

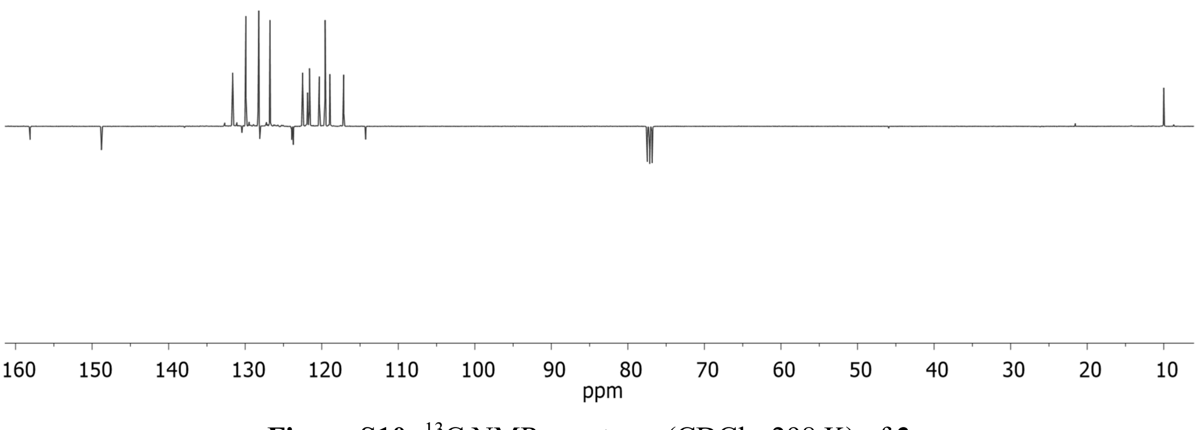

Figure S10: ^{13}C NMR spectrum ($CDCl_3$, 298 K) of **3e**.

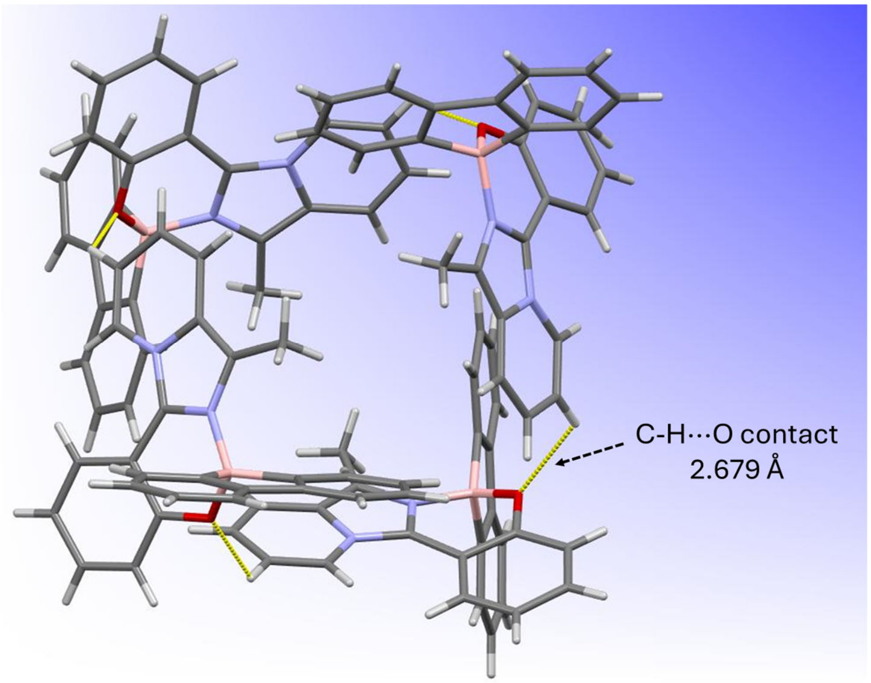

Figure S11. C-H···O contacts (yellow dashed lines) among four molecules of **3a** in the crystal packing.

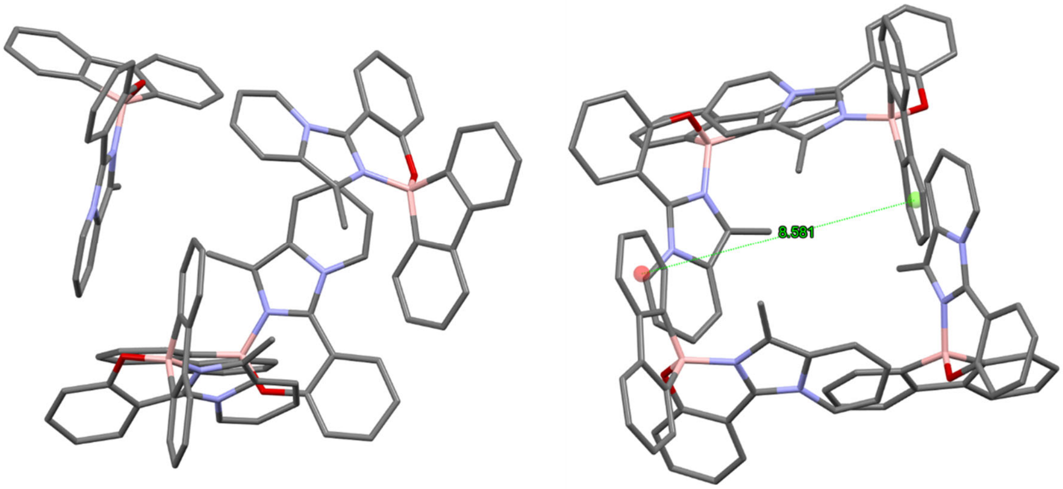

Figure S12. Crystal packing of **3a** seen from other orientations.

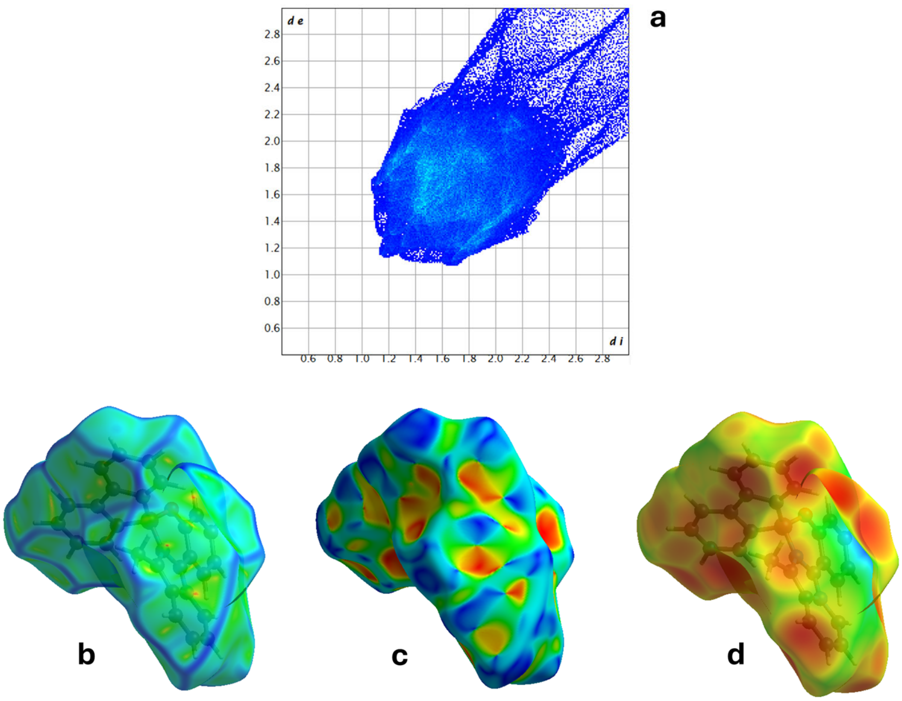

Figure S13. Fingerprint plot (a) and Hirshfeld surfaces of **3a**, mapped with curvedeness (b), shape index (c) and d_e (d) functions.

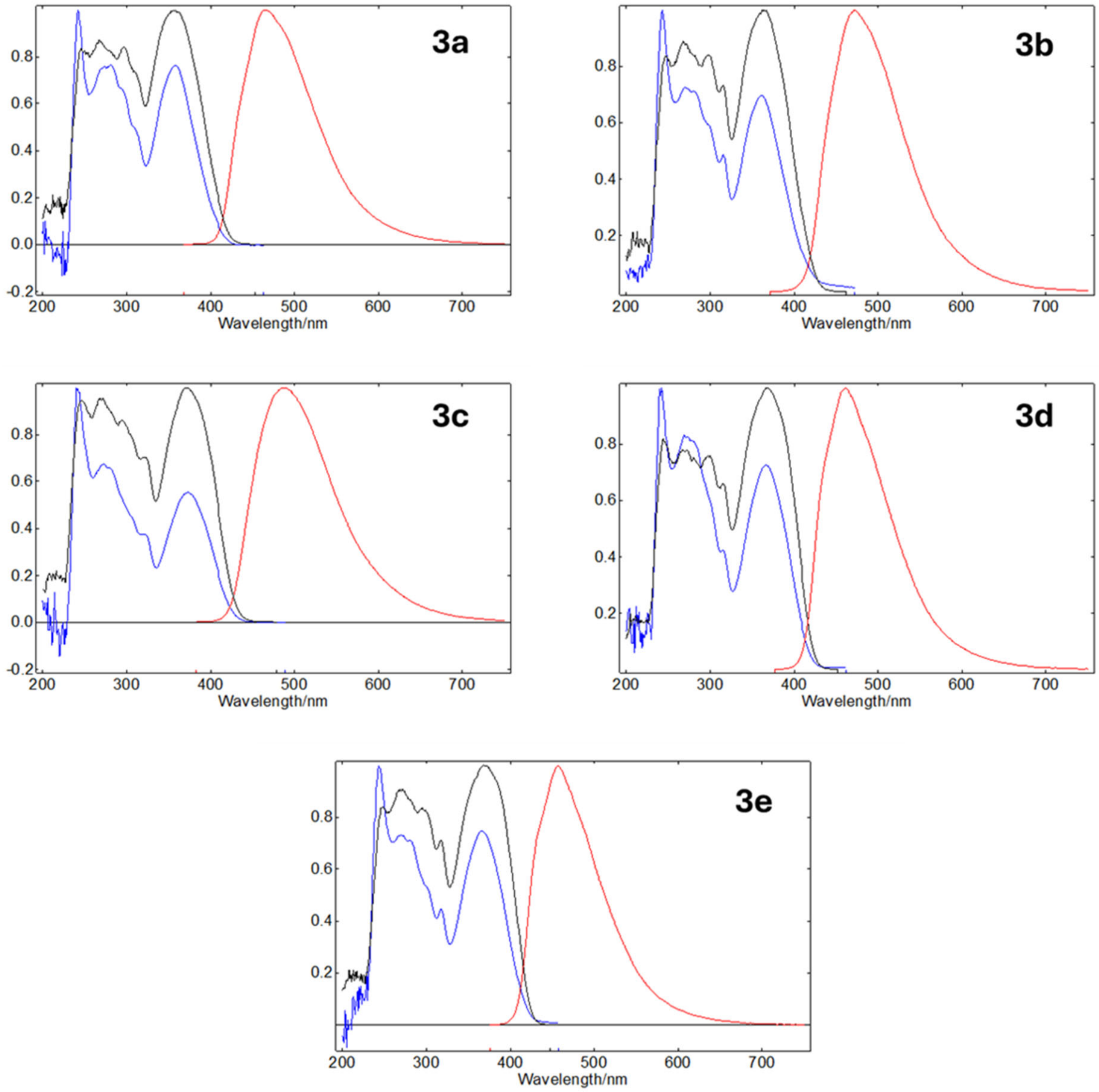

Figure S14: Absorption (blue), emission (red) and excitation (black) spectra of boron compounds **3a-e** recorded in dichloromethane solution (5·10^{-5} M).

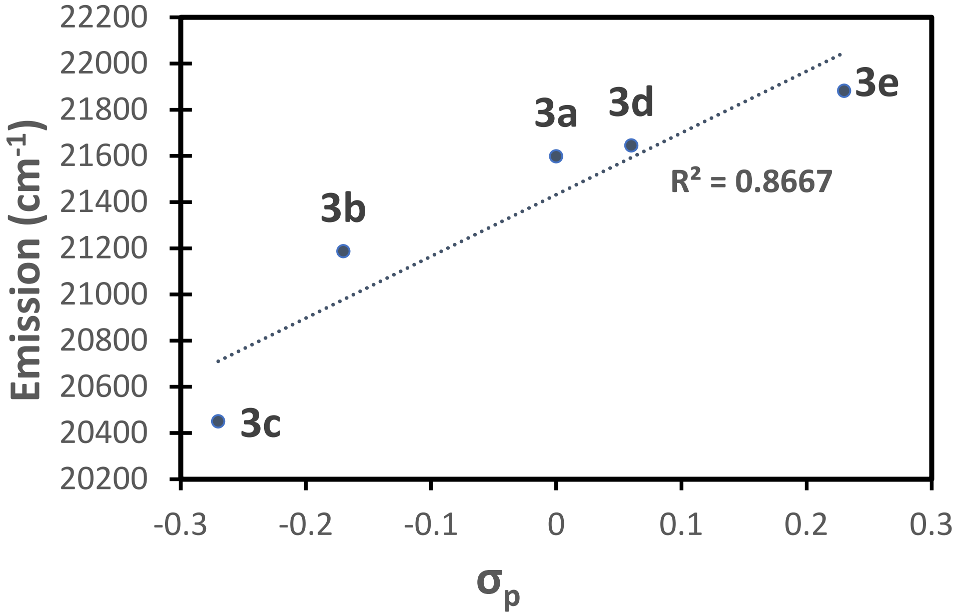

Figure S15: Correlation between fluorescence emission in solution and σ_p Hammett's constant of substituent R.

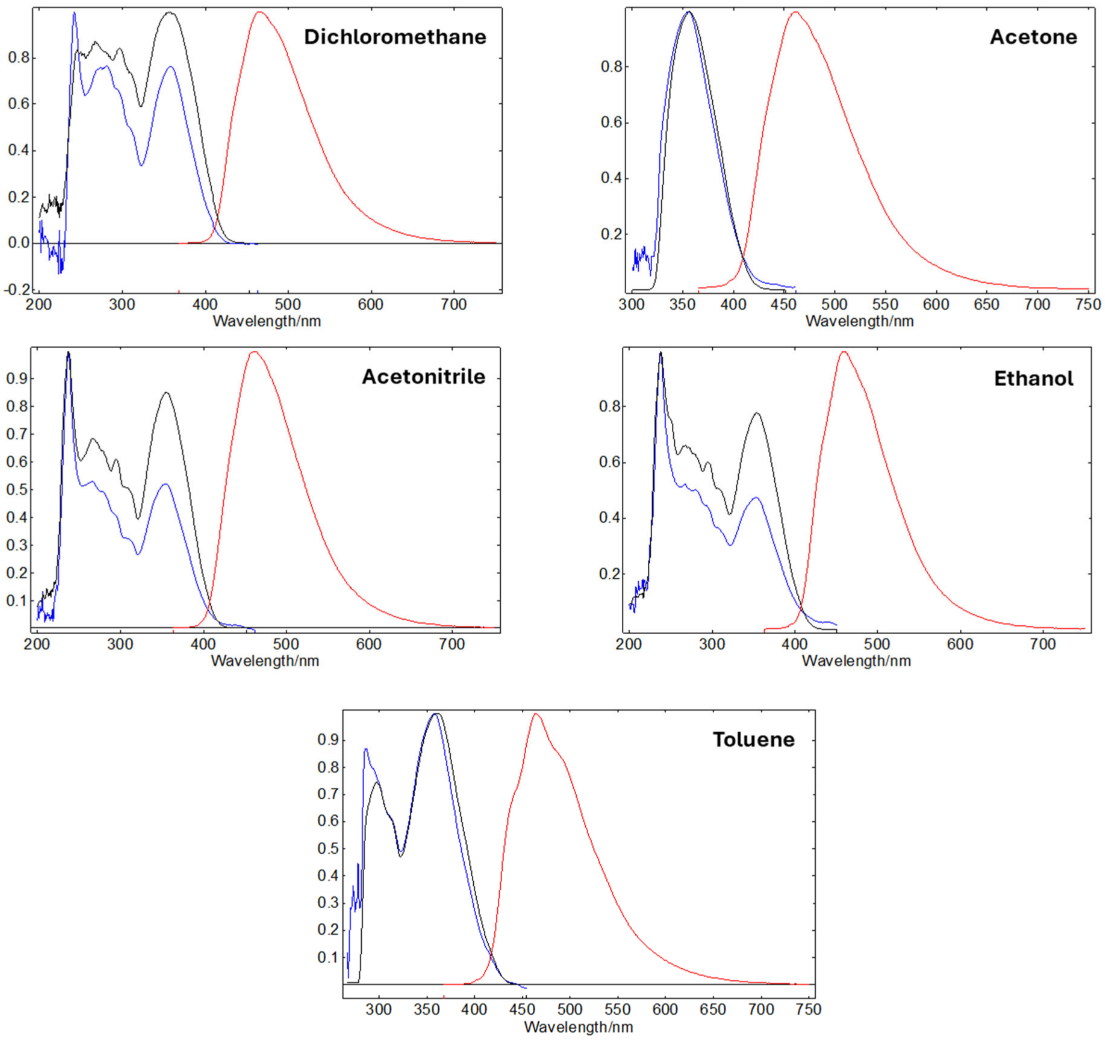

Figure S16: Normalized absorption (blue), emission (red) and excitation (black) spectra of compound **3a** recorded in various solvents ($5 \cdot 10^{-5}$ M).

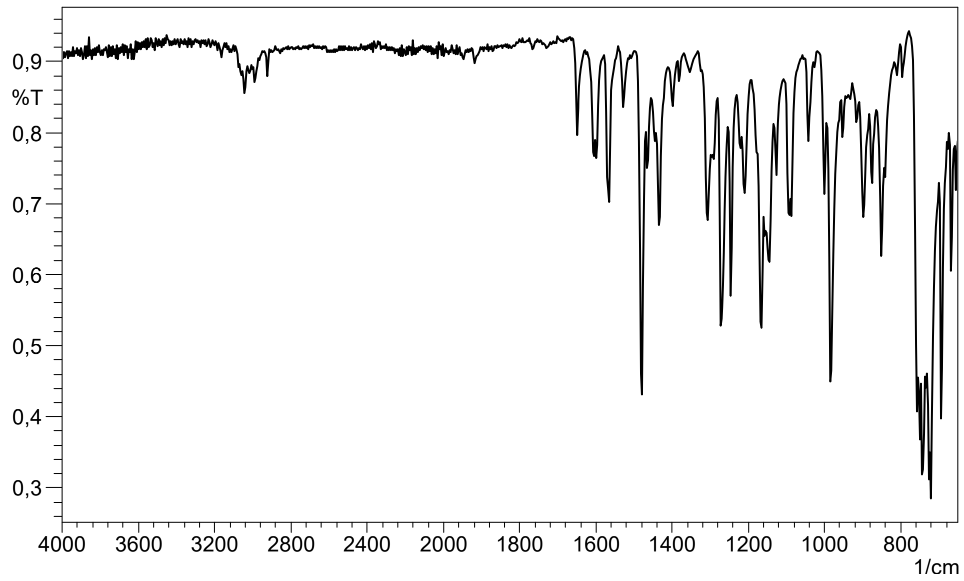

Figure S17: IR spectrum (ATR) of **3a**.

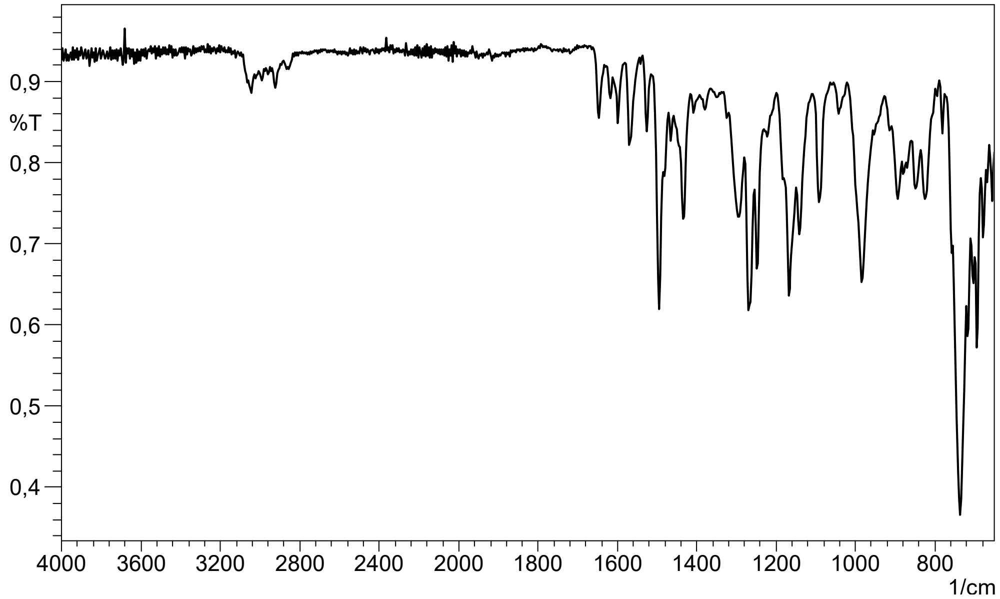

Figure S18: IR spectrum (ATR) of **3b**.

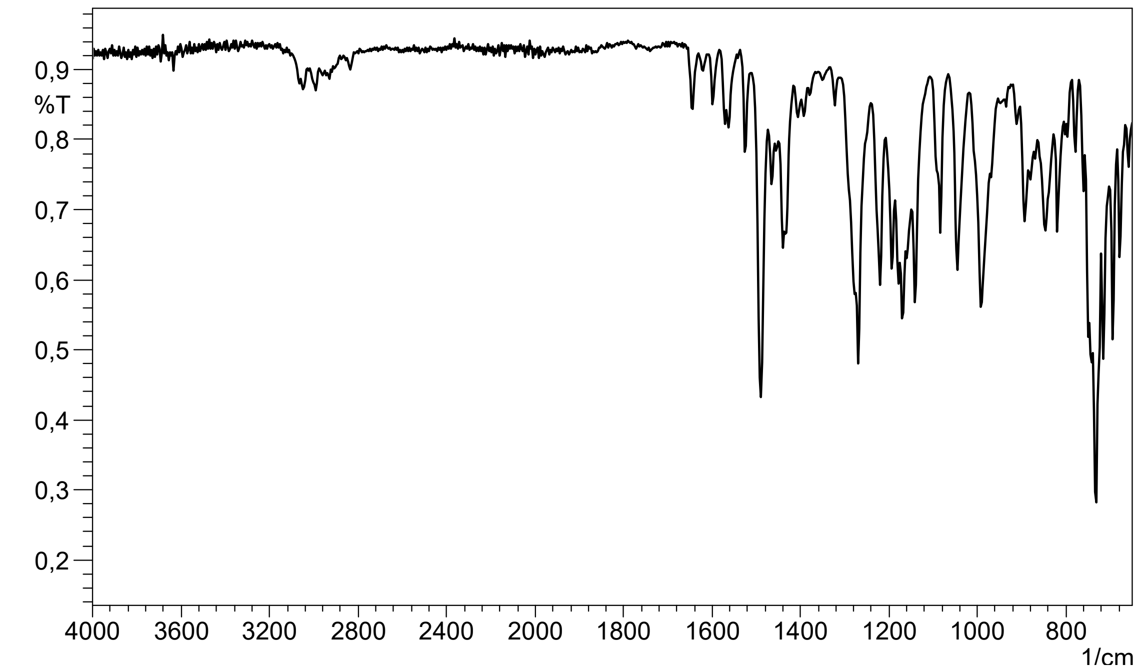

Figure S19: IR spectrum (ATR) of **3c**.

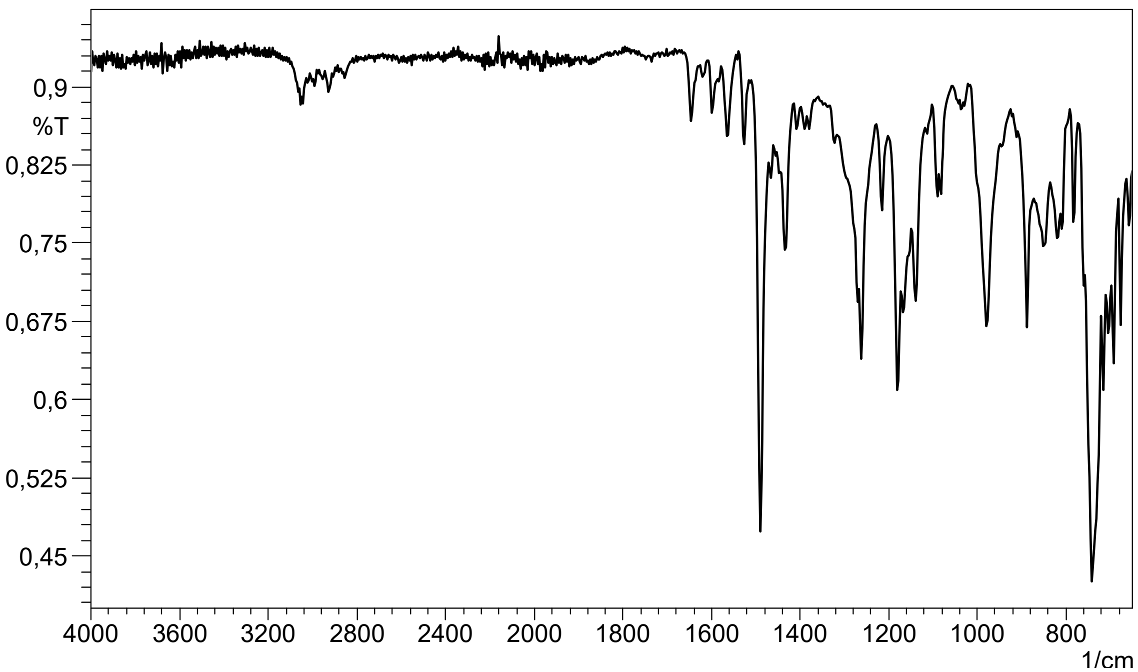

Figure S20: IR spectrum (ATR) of **3d**.

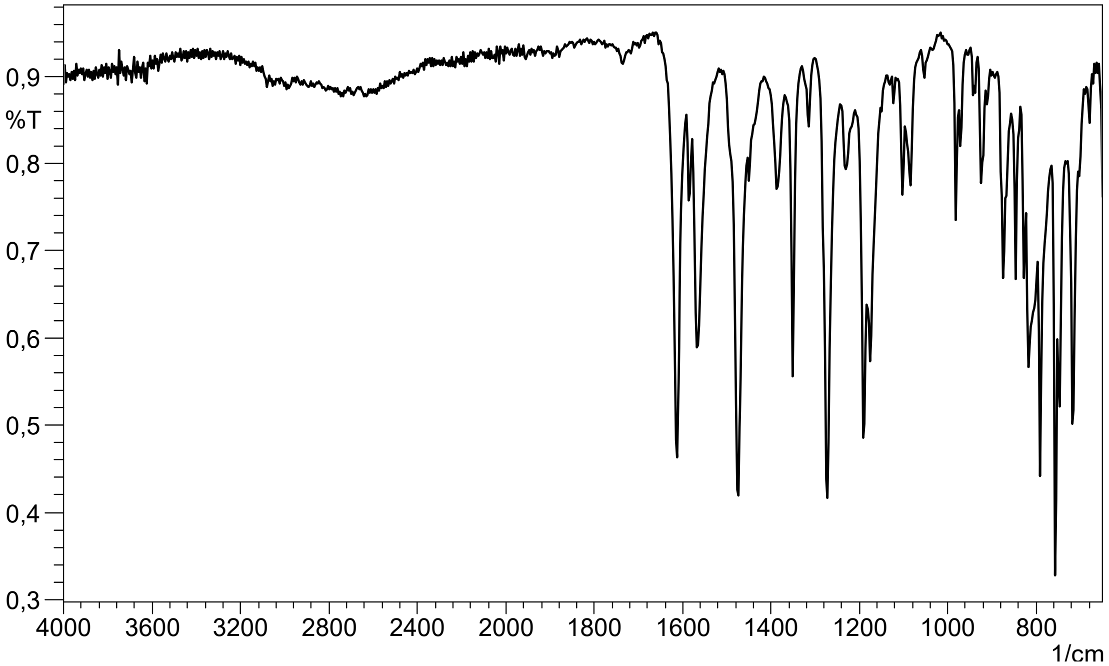

Figure S21: IR spectrum (ATR) of **3e**.

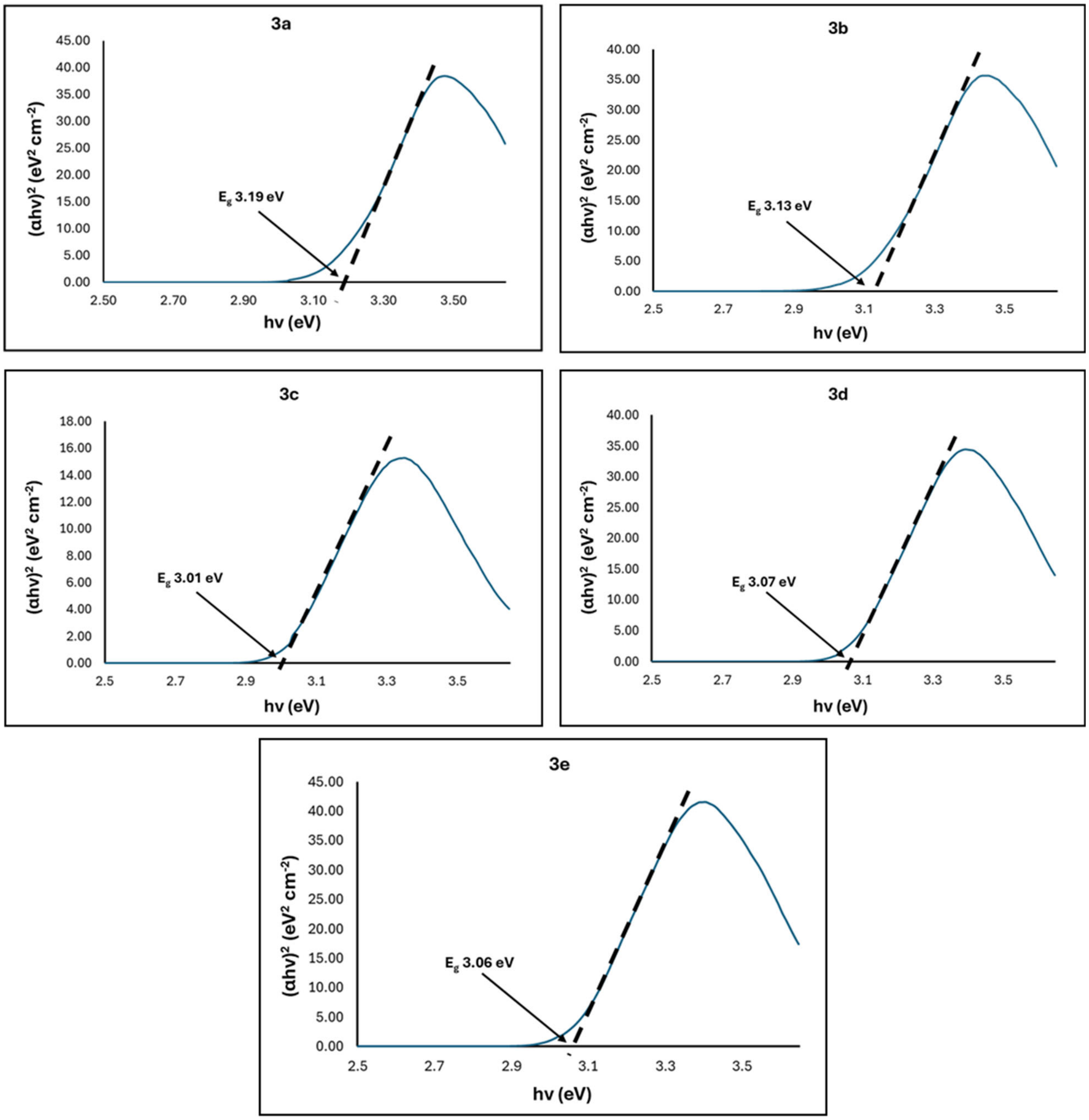

Figure S22: Tauc plots of compounds **3a-e** (CH_2Cl_2, $5{\cdot}10^{-5}$ M) with the respective band gap energy estimation.

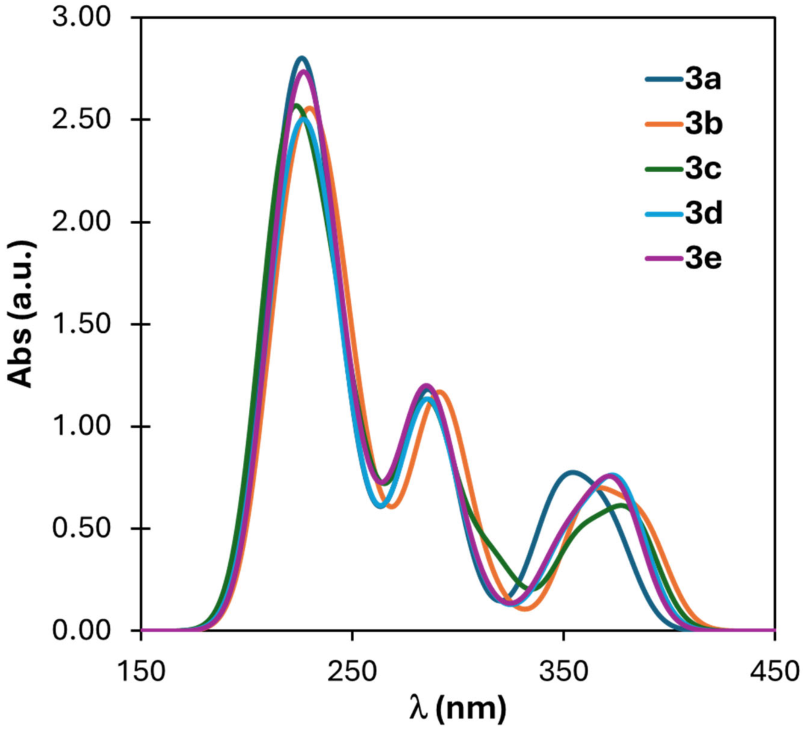

Figure S23: Calculated UV-vis spectra of compounds **3a-e**.

Table S1. Crystallographic and structure refinement parameters for compound **3a**.

	**3a** · H_2O
Chemical formula	$C_{26}H_{21}BN_2O_2$
Formula weight	404.293
Crystal system	Tetragonal
Space group	$I\,4_1/a$ (no. 88)
Crystal color and shape	Colorless prism
Crystal size	0.37 x 0.18 x 0.08
a (Å)	26.6167(5)
b (Å)	26.6167(5)
c (Å)	11.7810(2)
α (°)	90
β (°)	90
γ (°)	90
V (Å^3)	8346.2(3)
Z	16
T (K)	250(2)
D_c (g·cm^{-3})	1.287
μ (mm^{-1})	0.642
Scan range (°)	$7.08 < \theta < 69.74$
Unique reflections	3814
Observed refls [I>2σ(I)]	3346
R_{int}	0.0388
Final R indices [I>2σ(I)]*	0.0421, wR_2 0.1220
R indices (all data)	0.0467, wR_2 0.1169
Goodness-of-fit	1.0077
Max, Min Δρ/e (Å^{-3})	0.1482, - 0.1643

* Structures were refined on F_0^2: $wR_2 = [\square[w\,(F_0^2 - F_c^2)^2] / \square w\,(F_0^2)^2]^{1/2}$, where $w^{-1} = [\square(F_0^2) + (aP)^2 + bP]$ and $P = [\max(F_0^2, 0) + 2F_c^2]/3$

Table S2: Photophysical data of compound **3a** recorded in various solvents ($5 \cdot 10^{-5}$ M).

Solvent	λ_{abs} (nm)	λ_{exc} (nm)	λ_{em} (nm)	Stokes (eV)	φ_{PL}	τ (ns)
**Dichloromethane**	358	357	463	0.81	0.20	3.2
**Acetone**	355	356	461	0.80	0.18	2.9
**Acetonitrile**	354	355	462	0.81	0.20	3.1
**Ethanol**	353	354	460	0.82	0.21	3.2
**Toluene**	358	362	464	0.79	0.17	3.0

Table S3: Benchmark DFT calculations for compound **3a**'s HOMO-LUMO band gap.

**Optical Eg (eV)****	**Calculated Eg (eV)***							
	B3LYP-D3(BJ)	MO6	PBE-B3(BJ)	SCAN-B3(BJ)	PBE0	LDA	CAM-B3LYP	PBE0 (opt) / B2PLYP (sp)
3.19	3.86	4.31	2.51	2.76	4.22	2.48	6.37	6.01
error %	21	35	-21	-13	32	-22	100	88

Table S4: HOMO-LUMO band gap (eV) of compounds **3a-e** calculated from the ionization potential and electron affinity.

	**Optical Eg**	**PBE-D3(BJ) (IP/ EA)**	**error %**
**3a**	3.19	3.15	-1.3
**3b**	3.13	3.08	-1.6
**3c**	3.01	2.95	-2.0
**3d**	3.07	3.06	-0.3
**3e**	3.06	3.08	0.7
