# Supplementary material for: Boron-Centered Compounds: Exploring the Optical Properties of Spiro Derivatives with Imidazo[1,5-a]Pyridines
Source: Molecules. 2025 Jun 11;30(12):2552. doi: 10.3390/molecules30122552 (PMC12195987; doi:10.3390/molecules30122552)

# checkCIF/PLATON report

Structure factors have been supplied for datablock(s) compound3a

THIS REPORT IS FOR GUIDANCE ONLY. IF USED AS PART OF A REVIEW PROCEDURE FOR PUBLICATION, IT SHOULD NOT REPLACE THE EXPERTISE OF AN EXPERIENCED CRYSTALLOGRAPHIC REFEREE.

No syntax errors found. CIF dictionary Interpreting this report

# Datablock: compound3a

---

Bond precision: C-C = 0.0023 A Wavelength=1.54186

| | | | |
|---|---|---|---|
| Cell: | a=26.6167(5) | b=26.6167(5) | c=11.7810(2) |
| | alpha=90 | beta=90 | gamma=90 |
| Temperature: | 250 K | | |

| | Calculated | Reported |
|---|---|---|
| Volume | 8346.2(3) | 8346.2(3) |
| Space group | I 41/a | I 41/a |
| Hall group | -I 4ad | -I 4ad |
| Moiety formula | C26 H19 B N2 O [+ solvent] | C26 H19 B N2 O, 1(H2O) |
| Sum formula | C26 H19 B N2 O [+ solvent] | C26 H21 B1 N2 O2 |
| Mr | 386.24 | 404.29 |
| Dx,g cm-3 | 1.230 | 1.287 |
| Z | 16 | 16 |
| Mu (mm-1) | 0.583 | 0.642 |
| F000 | 3232.0 | 3402.9 |
| F000' | 3240.91 | |
| h,k,lmax | 32,32,14 | 31,31,13 |
| Nref | 3941 | 3814 |
| Tmin,Tmax | 0.871,0.950 | 0.807,0.955 |
| Tmin' | 0.789 | |

Correction method= # Reported T Limits: Tmin=0.807 Tmax=0.955
AbsCorr = MULTI-SCAN

Data completeness= 0.968 Theta(max)= 69.740

R(reflections)= 0.0421( 3346) wR2(reflections)= 0.1220( 3814)

S = 1.054 Npar= 272

The following ALERTS were generated. Each ALERT has the format
**test-name_ALERT_alert-type_alert-level.**
Click on the hyperlinks for more details of the test.

## Alert level C

```
PLAT230_ALERT_2_C Hirshfeld Test Diff for    C17      --C18      .        5.4 s.u.
PLAT410_ALERT_2_C Short Intra H...H Contact  H6       ..H9       .       1.97 Ang.
                                                      x,y,z  =      1_555 Check
PLAT906_ALERT_3_C Large K Value in the Analysis of Variance ......      2.154 Check
PLAT910_ALERT_3_C Missing # of FCF Reflection(s) Below Theta(Min).          7 Note
                0  2  0,   2  2  0,   0  4  0,   0  1  1,  -1  2  1,   1  2  1,
                0  3  1,
PLAT911_ALERT_3_C Missing FCF Refl Between Thmin & STh/L=    0.600         34 Report
              -18 26  0,  18 26  0, -14 28  0,  14 28  0, -10 30  0,   8 30  0,
               10 30  0, -15 28  1,  15 28  1,  -6 31  1,  -4 31  1,   6 31  1,
               14 28  2,  11 29  2,   0  1 11,   0  0 12,   1  1 12,   0  2 12,
                0  1 13,  -1  2 13,   1  2 13,  -2  3 13,   0  3 13,   2  3 13,
               -1  4 13,   1  4 13,   0  5 13,   1  6 13,  -8  9 13,   8  9 13,
               -7 10 13,   7 10 13,  -6 11 13,   6 11 13,
```

## Alert level G

```
FORMU01_ALERT_2_G  There is a discrepancy between the atom counts in the
            _chemical_formula_sum and the formula from the _atom_site* data.
            Atom count from _chemical_formula_sum:C26 H21 B1 N2 O2
            Atom count from the _atom_site data:  C26 H19 B1 N2 O1
CELLZ01_ALERT_1_G Difference between formula and atom_site contents detected.
CELLZ01_ALERT_1_G ALERT: Large difference may be due to a
            symmetry error - see SYMMG tests
           From the CIF: _cell_formula_units_Z   16
           From the CIF: _chemical_formula_sum  C26 H21 B1 N2 O2
           TEST: Compare cell contents of formula and atom_site data
```

| atom | Z*formula | cif sites | diff |
|---|---|---|---|
| C | 416.00 | 416.00 | 0.00 |
| H | 336.00 | 304.00 | 32.00 |
| B | 16.00 | 16.00 | 0.00 |
| N | 32.00 | 32.00 | 0.00 |
| O | 32.00 | 16.00 | 16.00 |

```
PLAT041_ALERT_1_G Calc. and Reported SumFormula    Strings  Differ     Please Check
              Calc: C26 H19 B N2 O
              Rep.: C26 H21 B1 N2 O2
PLAT042_ALERT_1_G Calc. and Reported MoietyFormula Strings  Differ     Please Check
              Calc: C26 H19 B N2 O
              Rep.: C26 H19 B N2 O, 1(H2O)
PLAT051_ALERT_1_G Mu(calc) and Mu(CIF) Ratio Differs from 1.0 by .       9.18 %
PLAT073_ALERT_1_G H-atoms ref, but _hydrogen_treatment Reported as     constr Check
PLAT178_ALERT_4_G The CIF-Embedded .res File Contains SIMU Records          1 Report
PLAT605_ALERT_4_G Largest Solvent Accessible VOID in the Structure        162 A**3
PLAT769_ALERT_4_G CIF Embedded Explicitly Supplied Scattering Data     Please Note
PLAT860_ALERT_3_G Number of Least-Squares Restraints ............         330 Note
PLAT868_ALERT_4_G ALERTS Due to the Use of _smtbx_masks Suppressed          ! Info
PLAT912_ALERT_4_G Missing # of FCF Reflections Above STh/L=  0.600         56 Note
PLAT960_ALERT_3_G Number of Intensities with I  <   - 2*sig(I) ...          1 Check
PLAT969_ALERT_5_G The 'Henn et al.' R-Factor-gap value ..........       7.365 Note
```

```
            Predicted wR2: Based on SigI**2  1.66 or SHELX Weight 11.57
PLAT978_ALERT_2_G Number C-C Bonds with Positive Residual Density.         0 Info
```

---

```
  0 ALERT level A = Most likely a serious problem - resolve or explain
  0 ALERT level B = A potentially serious problem, consider carefully
  5 ALERT level C = Check. Ensure it is not caused by an omission or oversight
 16 ALERT level G = General information/check it is not something unexpected

  6 ALERT type 1 CIF construction/syntax error, inconsistent or missing data
  4 ALERT type 2 Indicator that the structure model may be wrong or deficient
  5 ALERT type 3 Indicator that the structure quality may be low
  5 ALERT type 4 Improvement, methodology, query or suggestion
  1 ALERT type 5 Informative message, check
```

---

It is advisable to attempt to resolve as many as possible of the alerts in all categories. Often the minor alerts point to easily fixed oversights, errors and omissions in your CIF or refinement strategy, so attention to these fine details can be worthwhile. In order to resolve some of the more serious problems it may be necessary to carry out additional measurements or structure refinements. However, the purpose of your study may justify the reported deviations and the more serious of these should normally be commented upon in the discussion or experimental section of a paper or in the "special_details" fields of the CIF. checkCIF was carefully designed to identify outliers and unusual parameters, but every test has its limitations and alerts that are not important in a particular case may appear. Conversely, the absence of alerts does not guarantee there are no aspects of the results needing attention. It is up to the individual to critically assess their own results and, if necessary, seek expert advice.

**Publication of your CIF in IUCr journals**

A basic structural check has been run on your CIF. These basic checks will be run on all CIFs submitted for publication in IUCr journals (*Acta Crystallographica*, *Journal of Applied Crystallography*, *Journal of Synchrotron Radiation*); however, if you intend to submit to *Acta Crystallographica Section C* or *E* or *IUCrData*, you should make sure that full publication checks are run on the final version of your CIF prior to submission.

**Publication of your CIF in other journals**

Please refer to the *Notes for Authors* of the relevant journal for any special instructions relating to CIF submission.

---

**PLATON version of 13/05/2024; check.def file version of 04/05/2024**

**Datablock compound3a** - ellipsoid plot

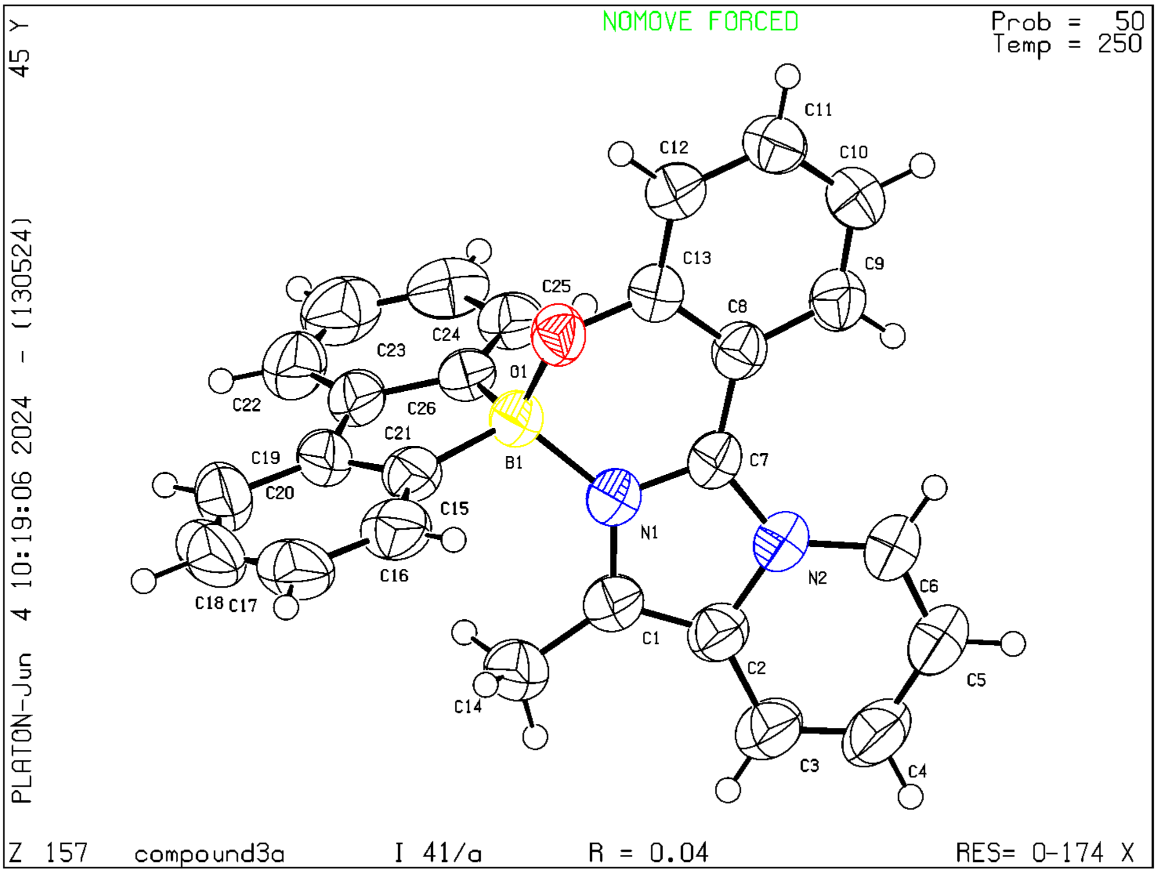

Supplement: Supplementary file 1 [file molecules-30-02552-s001.zip › checkcif_compound 3a.pdf]
